# Intranasal HD-Ad-FS vaccine induces systemic and airway mucosal immunities against SARS-CoV-2 and systemic immunity against SARS-CoV-2 variants in mice and hamsters

**DOI:** 10.3389/fimmu.2024.1430928

**Published:** 2024-08-30

**Authors:** Peter Zhou, Jacqueline Watt, Juntao Mai, Huibi Cao, Zhijie Li, Ziyan Chen, Rongqi Duan, Ying Quan, Anne-Claude Gingras, James M. Rini, Jim Hu, Jun Liu

**Affiliations:** ^1^ Department of Molecular Genetics, Faculty of Medicine, University of Toronto, Toronto, ON, Canada; ^2^ Translational Medicine Program, Hospital for Sick Children Research Institute, Toronto, ON, Canada; ^3^ Department of Laboratory Medicine and Pathobiology, University of Toronto, Toronto, ON, Canada; ^4^ Lunenfeld-Tanenbaum Research Institute, Mount Sinai Hospital, Toronto, ON, Canada; ^5^ Department of Biochemistry, Faculty of Medicine, University of Toronto, Toronto, ON, Canada

**Keywords:** COVID-19, HD-Ad, adenoviral vector, intranasal delivery, SARS-CoV-2, vaccine

## Abstract

The outbreak of coronavirus disease 19 (COVID-19) has highlighted the demand for vaccines that are safe and effective in inducing systemic and airway mucosal immunity against the aerosol transmission of severe acute respiratory syndrome coronavirus 2 (SARS-CoV-2). In this study, we developed a novel helper-dependent adenoviral vector-based COVID-19 mucosal vaccine encoding a full-length SARS-CoV-2 spike protein (HD-Ad-FS). Through intranasal immunization (single-dose and prime-boost regimens), we demonstrated that the HD-Ad-FS was immunogenic and elicited potent systemic and airway mucosal protection in BALB/c mice, transgenic ACE2 (hACE2) mice, and hamsters. We detected high titers of neutralizing antibodies (NAbs) in sera and bronchoalveolar lavages (BALs) in the vaccinated animals. High levels of spike-specific secretory IgA (sIgA) and IgG were induced in the airway of the vaccinated animals. The single-dose HD-Ad-FS elicited a strong immune response and protected animals from SARS-CoV-2 infection. In addition, the prime-boost vaccination induced cross-reactive serum NAbs against variants of concern (VOCs; Beta, Delta, and Omicron). After challenge, VOC infectious viral particles were at undetectable or minimal levels in the lower airway. Our findings highlight the potential of airway delivery of HD-Ad-FS as a safe and effective vaccine platform for generating mucosal protection against SARS-CoV-2 and its VOCs.

## Introduction

Coronavirus disease 2019 (COVID-19) is caused by severe acute respiratory syndrome coronavirus 2 (SARS-CoV-2) and led to more than 670 million confirmed cases and 6.8 million deaths worldwide by 2022. SARS-CoV-2 is an airborne-transmitted disease that infects the mucosal surface of the respiratory tract (1, 2). Tremendous efforts and major progress have been made to develop effective vaccines to control the spread of SARS-CoV-2 and its variants. Approved COVID-19 vaccines include those that are based on nucleic acid (3, 4) subunit protein (5), inactivated whole-virus (6), and non-replicating virus (7, 8). These vaccines are administered intramuscularly (IM) and are effective at preventing severe disease and curbing the hospitalization rate (9, 10). The IM vaccines induce robust systemic immunity; however, they only elicit limited immunity on airway mucosal surfaces which are the primary site of viral entry, shedding, and transmission (11). Therefore, the development of vaccines that can induce airway mucosal immunity as well as systemic immunity is in need.

Mucosal vaccines can induce potent airway immune memory through intranasal or oral immunization (12, 13). Intranasal vaccination mimics a natural route of viral entry where the antigen is directly introduced at the epithelium to elicit a mucosal immune response. Mucosal vaccines prime airway resident memory T cells which respond stronger and faster than circulating memory T cells during viral infection (14). They also induce antibodies, especially secretory IgA (sIgA), on the mucosal surface which blocks viral entry (8). Moreover, mucosal vaccination generates systemic immunity through the production of serum neutralizing antibodies (NAbs) (15). To date, around twelve intranasal COVID-19 vaccines are in clinical trials. Among these are live-attenuated virus, non-replicating virus, and subunit protein vaccines (16).

The helper-dependent adenoviral (HD-Ad) vector is a non-replicating third generation adenoviral vector that can be used for developing intranasal vaccines. HD-Ad leads to robust antigen delivery in the airway (17), which allows for effective antigen presentation by antigen-presenting cells (APCs) during T cell priming. Additionally, HD-Ad is devoid of all viral genes except for the packaging signals and inverted terminal repeats (ITRs), thereby minimizing inflammation compared to the first and second generations adenoviral vectors (18). This reduces the anti-vector immune response, enabling prolonged antigen expression in the airway mucosa (19). Antigen expression can be sustained for more than 3 weeks in the mouse airway (17). Sustained antigen expression continuously primes T follicular helper cells and B cells in the geminal center and induces long-term immunity with NAb production (20). Intranasal delivery of HD-Ad has been demonstrated to be safe in different animal models (19, 21), as well as in clinical trials (22). Previously, we developed an HD-Ad intranasal vaccine expressing the receptor-binding domain (RBD) of SARS-CoV-2. We demonstrated that intranasal delivery of HD-Ad-RBD elicited strong immunity and protected mice from SARS-CoV-2 infection (23).

Here, we describe an intranasal COVID-19 vaccine based on an HD-Ad vector that encodes the full-length spike protein (FS) of SARS-CoV-2 (HD-Ad-FS). The spike protein binds to the host cell receptor, ACE2, through its RBD domain and mediates virus-cell fusion. The spike protein was chosen as the immunogen because it contains multiple epitopes, including those on the RBD, which can elicit NAb production (24). We examine the efficacy of single-dose HD-Ad-FS against the SARS-CoV-2 ancestral strain (SARS-CoV-2 hereafter). Since the Beta, Delta, and Omicron strains are the main variants of concern (VOC) (25–27), we also test the efficacy of prime-boost HD-Ad-FS against these VOCs. We find that intranasal vaccination with single-dose HD-Ad-FS induces robust systemic, and upper and lower airway mucosal immunity against SARS-CoV-2 in hACE2 mice and hamsters. Furthermore, the prime-boost HD-Ad-FS elicits systemic and lower airway mucosal immunity against SARS-CoV-2 VOCs in these animal models.

## Results

### Construction and expression of the HD-Ad-FS vaccine

**Figure 1:**
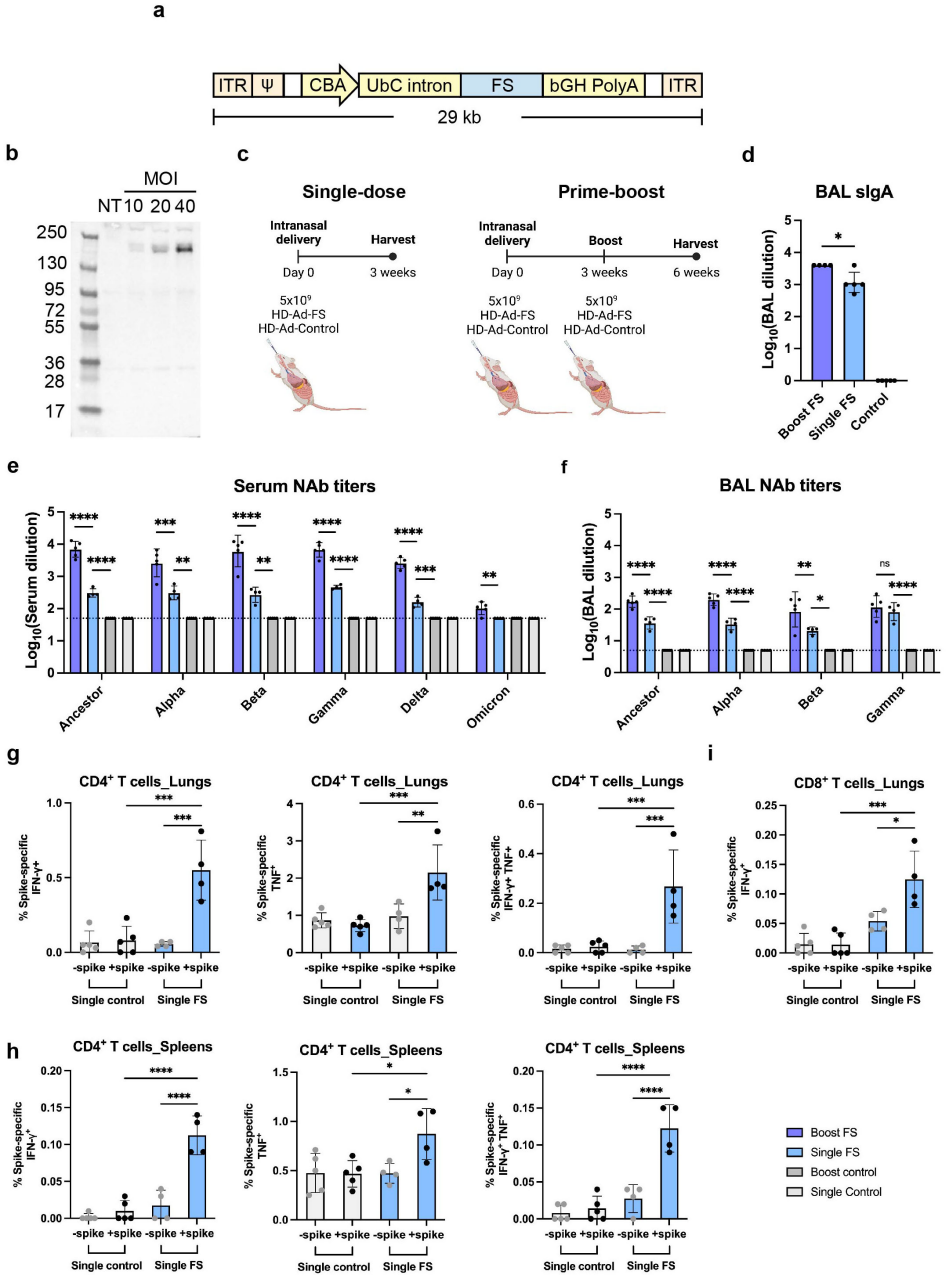
HD-Ad-FS induced efficient systemic and mucosal immunities in BALB/c mice. **(A)** Schematic of HD-Ad-FS vaccine. ITR, inverted terminal repeats; Ψ, adenoviral packaging signal; CBA, promoter of chicken beta-actin gene; UbC intron, first intron of ubiquitin C gene; FS, full-length spike gene of SARS-CoV-2 ancestral strain; bGH PolyA, polyadenylation tail of bovine growth hormone gene. **(B)** Western blot analysis of FS protein expression. IB3-1 cells were transduced with HD-Ad-FS at the indicated MOI, and proteins were harvested at day 3 post-transduction. **(C)** Schematic timelines of BALB/c mouse experiments. BALB/c mice were intranasally administered with HD-Ad-FS or HD-Ad-control at single-dose (5x10^9^ vp) and prime-boost regimens (5x10^9^ + 5x10^9^ vp, three weeks interval). Three weeks after the last administration, animal samples were harvested. **(D)** Levels of FS-specific sIgA responses in BALs were determined with Enzyme-linked immunosorbent assay (ELISA). The starting dilution factor was 1:1. **(E, F)** Neutralization of live SARS-CoV-2 and VOCs infection by sera **(E)** and BALs **(F)**. The Omicron used in this study was BA.1.18. The starting dilution factors were 1:50 and 1:5 for sera and BALs, respectively. **(G)** CD4^+^ T cell responses in the lungs of single-dose mice were measured by the production of IFN-γ (left), TNF (middle), and double-positive IFN-γ and TNF (right) at 3 weeks post-immunization, following *ex vivo* stimulation with spike antigen. **(H)** The production of IFN-γ (left), TNF (middle), and double-positive IFN-γ and TNF (right) in CD4^+^ T cells in the spleens of single-dose mice were measured at 3 weeks post-immunization, following *ex vivo* stimulation with spike antigen. **(I)** IFN-γ producing CD8^+^ T cells in the lungs of single-dose mice were measured by *ex vivo* stimulation with spike antigen. In all figures, dots represent individual mice (n=4 or 5). The dotted lines represent the limit of detection (LOD) of the assays. Statistical analyses were performed by one-way ANOVA. Bars and errors represent the geometric mean with geometric SD. *p<0.05, **p<0.01, ***p<0.001, ****p<0.0001, and ns, not significant. Data represent one independent animal experiment with indicated biological replicates.

The sequence of the full-length spike glycoprotein (FS) was codon-optimized, and two proline substitutions were introduced at positions 986 and 987 to stabilize the prefusion conformation (28). The expression of FS was driven by a chicken beta actin (CBA) promoter, and the first intron of human ubiquitin C (UbC) was included to increase the stability of the FS mRNA (**Figure 1A**). The transcription was terminated by a bovine growth hormone (bGH) polyadenylation tail.

To examine FS protein expression, human airway epithelial cells (IB3-1) were transduced with HD-Ad-FS at 10, 20, and 40 multiplicity of infection (MOI). Dose-dependent expression of the FS protein was detected from cell lysates by Western blot analysis at day 3 post-transduction, indicating that the FS protein was efficiently expressed (**Figure 1B**).

### HD-Ad-FS elicits robust systemic and airway mucosal immunity in BALB/c mice

To study the immunogenicity of HD-Ad-FS, we immunized BALB/c mice (n=5/group) with HD-Ad-FS via the intranasal route using single-dose (single-FS) and prime-boost (boost-FS) regimens (**Figure 1C**). For the single-FS group, mice were immunized with one dose of 5x10^9^ viral particles (vp). For the boost-FS group, mice received two doses of 5x10^9^ vp at a three-week interval. For the sham control mice, an HD-Ad empty vector (HD-Ad-control; single-control and boost-control) was used at the same doses and time points. Three weeks after the last vaccination, mice were euthanized, and bronchoalveolar lavages (BALs) and sera were collected. We chose 5x10^9^ vp for both single-dose and prime-boost regimens because this dosage had been shown to induce robust immune protection against SARS-CoV-2 infection with a HD-Ad vector expressing RBD in our previous study (23).

We measured the FS-specific secretory IgA (sIgA) level in BAL by enzyme-linked immunosorbent assay (ELISA). High levels of sIgA were detected in BALs of the mice vaccinated with single-FS, with a further increase observed after the booster dose (**Figure 1D**), indicating that both regimens induced sIgA in the airway. The reciprocal geometric mean titers (GMTs) of sIgA were 1148.7 and 3981 in single-FS and boost-FS mice, respectively (**Figure 1D**). FS-specific sIgA was not detectable in BALs from HD-Ad-control treated mice (**Figure 1D**).

The levels of NAbs against SARS-CoV-2 and the VOCs in sera and BALs were measured with a 50% tissue culture infectious dose (TCID_50_) neutralization assay. We observed that the single-FS induced NAbs against SARS-CoV-2 in both sera and BALs, and that the boost-FS significantly enhanced the effect (**Figures 1E, F**). Moreover, we found that both HD-Ad-FS regimens induced cross-reactive NAbs against the variants. In single-FS mice, the reciprocal GMTs for the serum NAbs against SARS-CoV-2, Alpha, Beta, Gamma, and Delta were 303.9, 306.5, 268.9, 456.6, and 158.5, respectively, while the titer of the NAb against Omicron (BA.1.18 used in this study) was undetectable (**Figure 1E**). Boost-FS mice had ~1.5 log-fold increase in serum NAb titers against SARS-CoV-2 and all the variants (**Figure 1E**). In BALs of single-FS mice, the NAb titers were 36.6 (SARS-CoV-2), 33.5 (Alpha), 20.8 (Beta), and 82.9 (Gamma, **Figure 1F**). The NAb titers in BALs of boost-FS mice were similarly increased by ~1 log-fold compared to single-FS mice against SARS-CoV-2 and the variants (**Figure 1F**). There were not enough BAL samples to test NAb levels against Delta and Omicron. Both sera and BALs from HD-Ad-control treated mice showed undetectable NAb levels. These results suggest that intranasal vaccination with HD-Ad-FS can elicit a robust NAb response in both sera and the airway.

We examined spike-specific T cell responses in the lungs and spleens of single-dose BALB/C mice three weeks post-immunization. In the lungs, single-FS induced significantly increased numbers of IFN-γ^+^ CD4^+^ and TNF^+^ (formerly known as TNF-α) CD4^+^ T cells compared to the control (**Figure 1G**). Spike-specific IFN-γ^+^ TNF^+^ double-positive CD4^+^ T cells were also identified in the lungs of single-FS mice (**Figure 1G**). In addition, in the spleens, single-FS significantly increased the populations of IFN-γ^+^ CD4^+^ and TNF^+^ CD4^+^ T cells (**Figure 1H**). Spike-specific IFN-γ^+^ TNF^+^ double-positive CD4^+^ T cells were also detected in the spleens of single-FS mice (**Figure 1H**). Moreover, we identified a significantly elevated number of IFN-γ^+^ CD8^+^ T cells (**Figure 1I**). These results indicated that in single-FS mice, Th1-type responses were induced in the lungs and spleens, and a cytotoxic CD8^+^ T cell response was elicited in the lungs.

### Single-dose HD-Ad-FS protects hACE2 mice from SARS-CoV-2 infection

**Figure 2:**
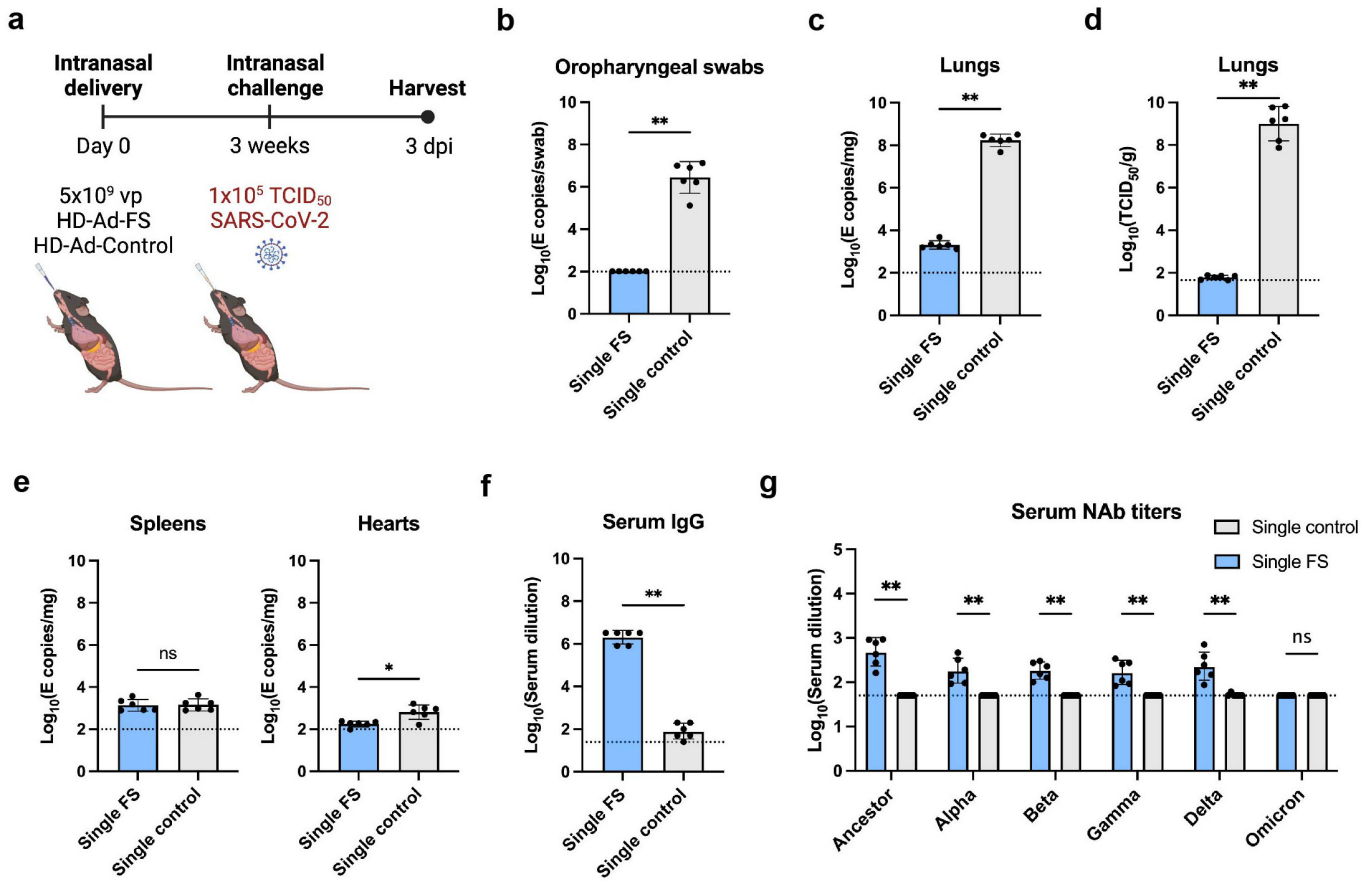
Vaccination of single-dose HD-Ad-FS protected hACE2 mice from SARS-Cov2 in the upper airway and lungs. **(A)** Schematic timeline of single-dose hACE2 experiment. The hACE2 mice were immunized with a single-dose of HD-Ad-FS (single-FS) or HD-Ad-control (single-control) at 5x10^9^ vp via intranasal delivery. Three weeks after immunization, the hACE2 mice were intranasally challenged with SARS-CoV-2 at 1x10^5^ TCID_50_. Animal samples were harvested at day 3 post-infection (dpi). **(B)** RNA levels of SARS-CoV-2 in oropharyngeal swabs were determined with RT-qPCR. **(C)** RNA levels of SARS-CoV-2 in the lungs were determined with RT-qPCR. **(D)** The titers of infectious SARS-CoV-2 in the lungs were measured with TCID_50_ assay. **(E)** RNA levels of SARS-CoV-2 in the spleens (left) and hearts (right) were measured with RT-qPCR. **(F)** FS-specific IgG response in serum were determined with ELISA. The starting dilution was 1:25. **(G)** Serum neutralizing activities against SARS-CoV-2 and VOCs were measured with neutralization assays. Dots represent individual mice (n=6). The dotted lines represent the LOD of the assays. For RT-qPCR, the LOD was set to 100 E copies/sample. Statistical analyses were performed by Mann-Whitney test, two-tailed. Bars and errors represent geometric mean with geometric SD. *p<0.05, **p<0.01, and ns, not significant. Data represent one independent animal experiment with indicated biological replicates.

The hACE2 mice express human angiotensin I-converting enzyme 2 (hACE2) under the cytokeratin 18 (K18) gene promoter, and they develop severe disease upon SARS-CoV-2 infection (29). To examine the protective efficacy of single-dose HD-Ad-FS, we intranasally immunized hACE2 mice (n=12) with HD-Ad-FS (single-FS) or sham HD-Ad-control (single-control) at a dose of 5x10^9^ vp. At day 21 post-vaccination, the immunized hACE2 mice were intranasally challenged with SARS-CoV-2 at 1x10^5^ TCID_50_. At day 3 post-infection (dpi), the hACE2 mice were euthanized, and serum, lung, spleen, and heart samples were harvested for analysis (**Figure 2A**).

The levels of viral RNA and infectious virus in the airway were measured with real-time quantitative PCR (RT-qPCR) and TCID_50_ assays, respectively. In single-FS hACE2 mice, the viral RNA was undetectable in oropharyngeal swabs (**Figure 2B**). In contrast, the viral RNA was at high levels (≥10^6^ copies/swab) in single-control hACE2 mice (**Figure 2B**). Moreover, the levels of viral RNA in the lungs were significantly lower in single-FS hACE2 mice (2.3x10^3^ copies/mg tissue) than in single-control mice (2.0x10^8^ copies/mg tissue, **Figure 2C**). Notably, the titers of infectious virus were undetectable in the lungs of single-FS hACE2 mice, while the titers were very high (2.6x10^9^ TCID_50_/g lung) in the lungs of single-control hACE2 mice (**Figure 2D**). These results show that SARS-CoV-2 could not replicate in the airway of single-FS vaccinated hACE2 mice. Additionally, there was no significant difference in the levels of viral RNA in the spleens of single-FS and single-control hACE2 mice, and the viral RNA in the hearts of single-FS hACE2 mice were undetectable or very low at 3 dpi (**Figure 2E**).

The titers of FS-specific IgG and NAb in serum were measured by ELISA and TCID_50_ assays, respectively. Significantly higher titers of IgG (reciprocal GMT 2.0x10^6^) were detected in single-FS hACE2 mice compared to single-control hACE2 mice (79.4, **Figure 2F**). In addition, the NAb titers were 480.9 (SARS-CoV-2), 182.6 (Alpha), 182.3 (Beta), 165.5 (Gamma), and 228.6 (Delta) in single-FS hACE2 mice, whereas the NAb titers were undetectable or very low in single-control hACE2 mice (**Figure 2G**). The titer of NAb against Omicron was undetectable in single-FS hACE2 mice. These findings suggests that intranasal HD-Ad-FS vaccination could elicit NAbs in the sera, effectively protecting hACE2 mice from SARS-CoV-2 infection.

### Prime-boost HD-Ad-FS protects the lungs of hACE2 mice from SARS-CoV-2 VOCs

**Figure 3:**
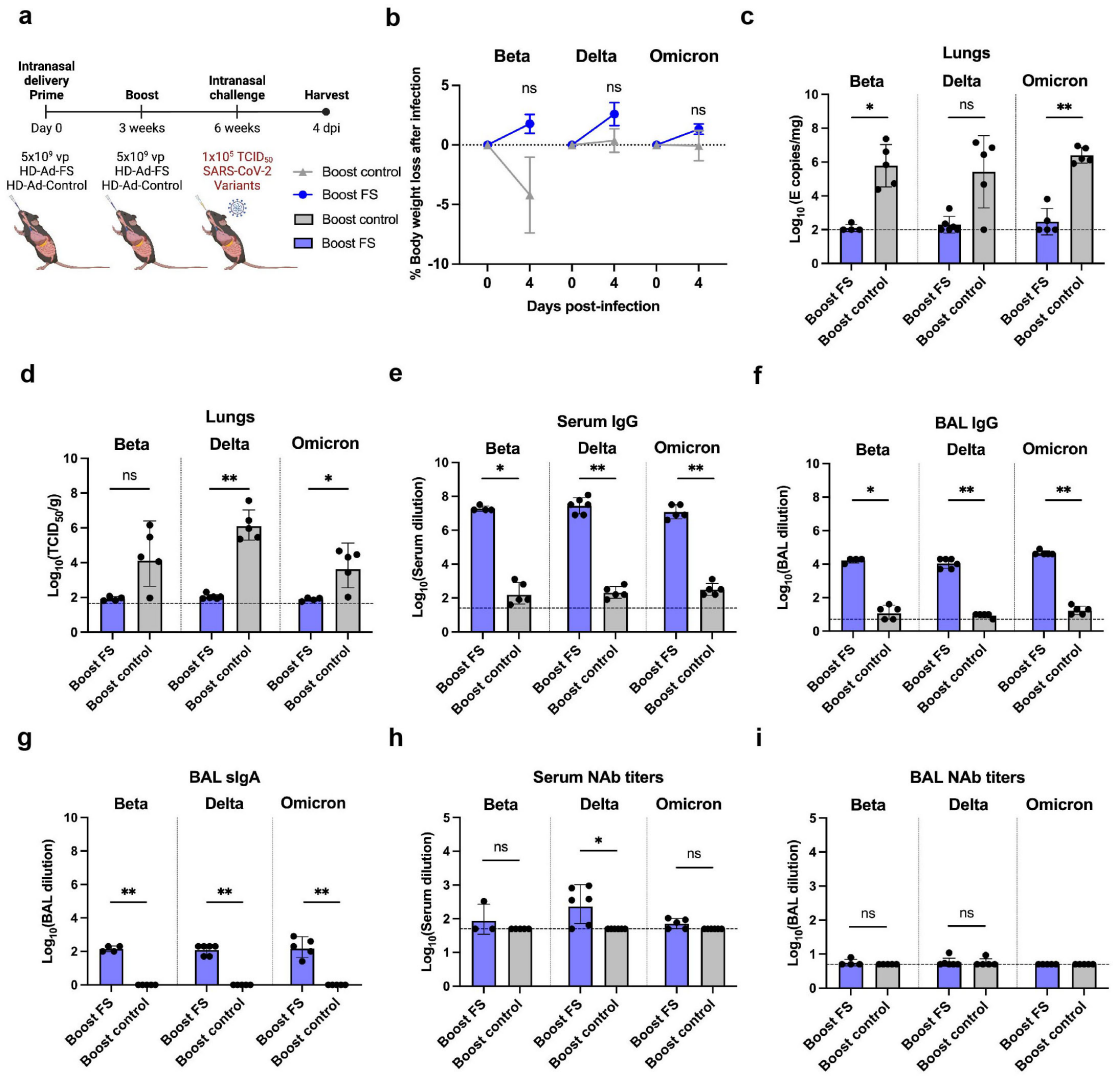
Prime-boost vaccination of HD-Ad-FS protected hACE2 mice from SARS-CoV-2 VOCs in the lungs. **(A)** Schematic timeline of prime-boost hACE2 experiment. The mice were intranasally administered with a prime-boost regimen of HD-Ad-FS or HD-Ad-control (5x10^9^ + 5x10^9^ vp, three-week interval). Three weeks after the boost dose, the mice were intranasally challenged with a SARS-CoV-2 variant (Beta, Delta, or Omicron) at 1x10^5^ TCID_50_. Animal samples were harvested at 4 dpi. **(B)** Body weight was recorded at 0 (pre-challenge) and 4 dpi after challenge. **(C)** SARS-CoV-2 variant RNA levels in the lungs were measured with RT-qPCR. **(D)** The titers of infectious SARS-CoV-2 variant in the lungs were determined with TCID_50_ assays. **(E, F)** The titers of FS-specific IgG in sera **(E)** and BALs **(F)** were determined with ELISA. The starting dilution factors were 1:25 and 1:5 for sera and BALs, respectively **(G)** The titers of FS-specific sIgA in BALs were measured with ELISA. The starting dilution factor was 1:1. **(H, I)** Neutralizing activities in sera **(H)** and BALs **(I)** against SARS-CoV-2 variants were determined with neutralization assays. Dots represent individual mice (n=4, 5, or 6). For **(C-F, H, I)**, the horizontal dotted lines represent the LOD of the assays. For **(B)**, statistical analyses were performed by two-way ANOVA. Error bars represent mean ± s.e.m. For **(C-I)**, statistical analyses were performed by Mann-Whitney test, two-tailed. Error bars represent geometric mean with geometric SD. *p<0.05, **p<0.01, and ns, not significant. Data represent one independent animal experiment with indicated biological replicates.

The antibody-escape mutations and deletions in VOCs, particularly in the Beta, Delta, and Omicron strains, has resulted in reduced sensitivity to the neutralizing antibody elicited by COVID-19 vaccines (25, 30, 31). Therefore, we aimed to investigate whether intranasal administration of HD-Ad-FS could confer protection against these variants. hACE2 mice (n=36, equal ratio of sex) were intranasally immunized with a prime-boost regimen (5x10^9^ + 5x10^9^ vp) of HD-Ad-FS (boost-FS) or HD-Ad-control (boost-control) at a three-week interval. Three weeks after the boost dose, the hACE2 mice were divided into three groups (n=12/group) and intranasally challenged with SARS-CoV-2 VOCs Beta, Delta, or Omicron at 1x10^5^ TCID_50_. At 4 dpi, the hACE2 mice were euthanized, and lung, serum, and BAL samples were harvested (**Figure 3A**). Due to anesthetic issues, some hACE2 mice (n=3 in Beta, n=1 in Delta, and n=2 in Omicron group) died immediately after challenging with the variants. Although, disease signs typically occur in hACE2 mice at 5 dpi we harvested tissues at 4 dpi. This allowed us to investigate the ability of HD-Ad-FS to control the viral replication during the initial stages of infection, before the onset of disease symptoms.

The body weights of hACE2 mice were recorded at 0 (pre-infection) and 4 dpi. In boost-FS hACE2 mice, they all gained weight from 0 to 4 dpi: 1.8% (Beta), 2.6% (Delta), and 1.3% (Omicron, **Figure 3B**). However, the weight difference was not statistically significant between boost-FS and boost-control hACE2 mice in each variant group (**Figure 3B**). Additionally, there was no significant difference in weight change between male and female hACE2 mice in each group (**Supplementary Figure 1**).

The levels of viral RNA and infectious virus in the lungs were measured with RT-qPCR and TCID_50_ assays, respectively, at 4 dpi. The viral RNA level was undetectable (3/4 in Beta, 4/6 in Delta, and 3/5 in Omicron group) or very low in the lungs of boost-FS hACE2 mice. In contrast, high levels of viral RNA (6.1x10^5^ in Beta, 1.1x10^5^ in Delta, and 2.5x10^6^ copies/mg in Omicron) were detected in the lungs of boost-control hACE2 mice (**Figure 3C**). In addition, the infectious virus was at very low levels in the lungs of boost-FS hACE2 mice, whereas high levels (2.5x10^4^ in Beta, 1.4x10^6^ in Delta, and 6.0x10^3^ TCID_50_/g lung in Omicron group) were detected in the lungs of boost-control hACE2 mice (**Figure 3D**). There was no significant difference in viral RNA levels in the lung between male and female mice in each VOC group (**Supplementary Figure 2A**). Furthermore, there was no difference in the levels of viral RNA from oropharyngeal swabs between boost-FS and boost-control hACE2 mice at 4 dpi (**Supplementary Figure 3**). These results suggest that prime-boost HD-Ad-FS protects against VOC infection in the lungs but not in the upper airway.

The levels of FS-specific IgG and sIgA were measured with ELISA at 4 dpi. In boost-FS hACE2 mice, the IgG titers were significantly elevated in sera and BALs, compared to boost-control hACE2 mice (**Figures 3E, F**). Specifically, in boost-FS hACE2 mice, the IgG reciprocal GMTs in sera were 1.9x10^7^ (Beta), 2.9x10^7^ (Delta), and 1.2x10^7^ (Omicron), and those in the BALs were 1.7x10^4^ (Beta), 1.1x10^4^ (Delta), and 4.6x10^4^ (Omicron). Notably, sIgA was also induced in the BALs of boost-FS hACE2 mice, with reciprocal GMTs of 141.4 (Beta), 126.0 (Delta), and 174.1 (Omicron, **Figure 3G**). There was no significant difference in the levels of sIgA between male and female mice in each variant group (**Supplementary Figure 4A**).

The titers of NAbs in sera and BALs were measured with the TCID_50_ neutralization assay at 4 dpi. Compared to boost-control hACE2 mice, serum NAbs were only significantly increased in Delta-challenged boost-FS hACE2 mice (**Figure 3H**). However, in all the VOC groups, the BAL NAbs were not increased significantly in boost-FS hACE2 mice compared to boost-control hACE2 mice (**Figure 3I**). There was no significant difference in either serum or BAL NAbs between male and female mice in each VOC group (**Supplementary Figures 4B, C**).

### Single-dose HD-Ad-FS protects hamsters from SARS-CoV-2 infection

**Figure 4:**
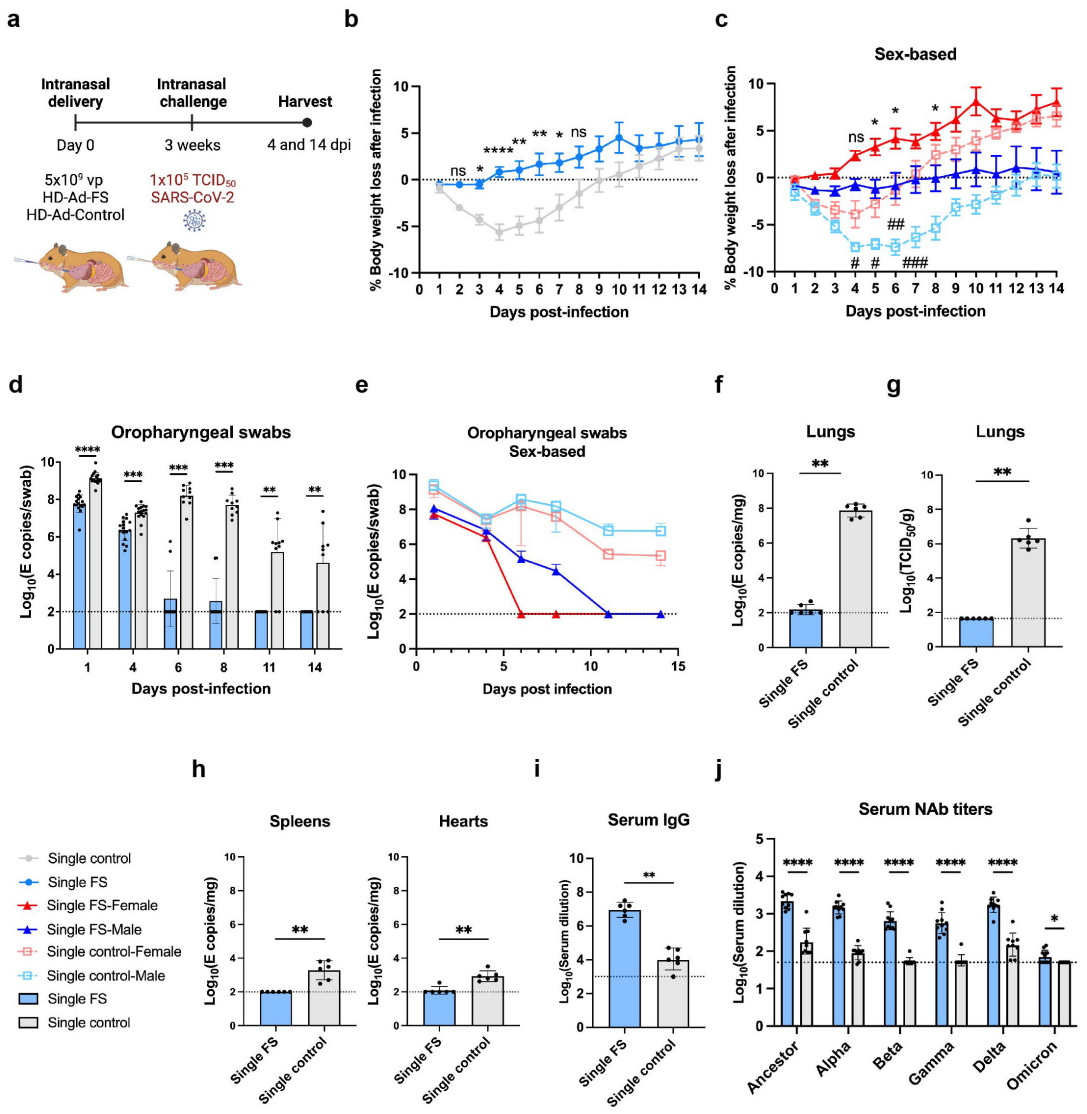
Single-dose vaccination of HD-Ad-FS protected hamsters against SARS-CoV-2 in the upper airway and lungs. **(A)** Schematic timeline of single-dose hamster experiment. Hamsters were immunized with a single-dose of HD-Ad-FS or HD-Ad-control at 5x10^9^ vp via intranasal route. Three weeks after immunization, hamsters were intranasally challenged with SARS-CoV-2 at 1x10^5^ TCID_50_. Animal samples were harvested at 4 and 14 dpi. **(B, C)** Body weight was monitored at the indicated days after challenging with SARS-CoV-2. For **(C)**, the change of weight in male and female hamsters were analyzed separately. **(D, E)** RNA levels of SARS-CoV-2 from oropharyngeal swabs were determined with RT-qPCR. Swabs were collected at the indicated time points. For **(E)**, the levels of viral RNA in male and female hamsters were analyzed separately. **(F)** RNA levels of SARS-CoV-2 in lungs were determined with RT-qPCR at 4 dpi. **(G)** The titers of infectious SARS-CoV-2 in lungs were determined with TCID_50_ assays at 4 dpi. **(H)** RNA levels of SARS-CoV-2 in spleens (left) and hearts (right) were measured with RT-qPCR at 4 dpi. **(I)** The titers of FS-specific IgG in sera were measured with ELISA at 4 dpi. The starting dilution factor was 1:1000. **(J)** Serum NAb activities against SARS-CoV-2 and variants were determined with neutralization assays at 14 dpi. Dots represent individual hamsters (4 dpi, n=6; 14 dpi, n=9 or 10). For **(D-J)**, the horizontal dotted lines represent the LOD of the assays. For **(B, C)**, statistical analyses were performed by two-way ANOVA; error bars represent mean ± s.e.m. For **(D, E)**, statistical analyses were performed by two-way ANOVA; error bars represent geometric mean with geometric SD. For **(F-J)**, statistical analyses were performed by Mann-Whitney test, two-tailed. Error bars represent geometric mean with geometric SD. For **(C)**, the asterisk signs represent statistical analyses in single-FS hamsters, and the pound signs represent statistical analyses in single-control hamsters. */# p<0.05, **/## p<0.01, ***<0.001, ****/####p<0.0001, and ns, not significant. Data represent one independent animal experiment with indicated biological replicates.

The protective efficacy of HD-Ad-FS was further examined in hamsters, which are susceptible to SARS-CoV-2 infection and can develop pathology similar to that of COVID-19 patients (32). The hamsters (n=32, equal ratio of sex) were intranasally immunized with a single dose of HD-Ad-FS (single-FS) or HD-Ad-control (single-control) at 5x10^9^ vp. At day 21 post-vaccination, the hamsters were intranasally challenged with SARS-CoV-2 at 1x10^5^ TCID_50_. Hamsters were euthanized at 4 (n=12) and 14 (n=20) dpi, and oropharyngeal swab, serum, lung, spleen, and heart samples were collected and analyzed (**Figure 4A**).

The body weight of hamsters was monitored for 14 days following SARS-CoV-2 infection and compared to their pre-infection weight. In single-FS hamsters, the average weight mildly decreased between 1 and 3 dpi but then increased and exceeded the pre-infection levels between 4 and 14 dpi (**Figure 4B**). However, single-control hamsters had sustained weight loss between 1 and 9 dpi (**Figure 4B**). Specifically, the most severe weight loss was 0.5% in single-FS hamsters at 3 dpi, as compared to 5.6% in single-control hamsters at 4 dpi (**Figure 4B**). The weight change observed in single-FS hamsters indicates the potential efficacy of HD-Ad-FS in preventing severe disease and promoting recovery following SARS-CoV-2 infection. Sex-based analysis showed that male hamsters in both single-FS and single-control groups had significantly more severe weight loss than female hamsters (**Figure 4C**).

Viral RNA levels in oropharyngeal swabs were measured with RT-qPCR. In single-FS hamsters, viral RNA was undetectable in 8 out of 10 swabs at 6 dpi and in all swabs at 11 dpi (**Figure 4D**). In contrast, in single-control hamsters, high levels of viral RNA (2.7x10^7^ copies/swab) were detected in all swabs at 6 dpi and the levels remained elevated in 8 out of 10 swabs at 11 dpi (**Figure 4D**). These findings indicated that single-FS could reduce SARS-CoV-2 infection and replication in the upper airway. In addition, sex-based analysis indicated that in female HD-Ad-FS hamsters viral RNA levels decreased more rapidly and became undetectable earlier than those in male hamsters (**Figure 4E**). In the single-control group, viral RNA levels were high in both male and female hamsters after infection, with male hamsters having slightly higher levels than female hamsters (**Figure 4E**).

The levels of viral RNA and infectious virus in the lungs were determined with RT-qPCR and TCID_50_ assays, respectively, at 4 dpi. The viral RNA was either undetectable (4/6) or very low (3.6x10^2^ copies/mg) in single-FS hamsters, whereas the viral RNA levels were high (9.5x10^7^ copies/mg) in all single-control hamsters (**Figure 4F**). Furthermore, infectious virus was undetectable in single-FS hamsters, whereas substantial levels of infectious virus were detected in all single-control hamsters (**Figure 4G**). These findings indicate that single-FS could effectively prevent SARS-CoV-2 infection and replication in the lungs. Sex-based analysis showed no significant difference in viral RNA (**Supplementary Figure 5A**) or infectious virus levels (**Supplementary Figure 5B**) between male and female hamsters.

The levels of viral RNA in the spleens and hearts were also measured with RT-qPCR at 4 dpi. Viral RNA was undetectable in the spleens and hearts of single-FS hamsters (5/6), but it was detectable in all single-control hamsters (**Figure 4H**). These findings indicate that single-FS could prevent systemic spread of SARS-CoV-2. Furthermore, sex-based analysis showed that male single-control hamsters had significantly higher levels of viral RNA in the spleens compared to female hamsters. However, there was no significant difference in the levels of viral RNA in the hearts between male and female hamsters in each treatment (**Supplementary Figure 5C**).

The levels of FS-specific IgG and NAbs in serum were measured with ELISA at 4 dpi and by TCID_50_ neutralization assays at 14 dpi, respectively. The level of serum IgG was increased significantly in single-FS hamsters (reciprocal GMTs 9.0x10^6^) compared to single-control hamsters (**Figure 4I**). Additionally, the NAb levels against SARS-CoV-2 were significantly higher in single-FS hamsters (ID_50_: 2184.3) compared to single-control hamsters (**Figure 4J**). Notably, in single-FS hamsters, the serum NAbs cross-reacted with the Alpha (1463.4), Beta (647.2), Gamma (562), Delta (1731.9), and Omicron VOCs (71.3, **Figure 4J**). The presence of serum IgG and NAbs indicate that intranasally delivered single-FS could elicit a systemic antibody response. There were no significant differences in the serum NAb levels between male and female hamsters in each treatment, except that the titers of female hamsters were significantly higher than those of male hamsters in the single-control group after SARS-CoV-2 infection (**Supplementary Figure 5D**). We did not measure sIgA levels in hamsters as there were no commercially available antibodies for hamster IgA at the time of the experiment.

**Figure 5:**
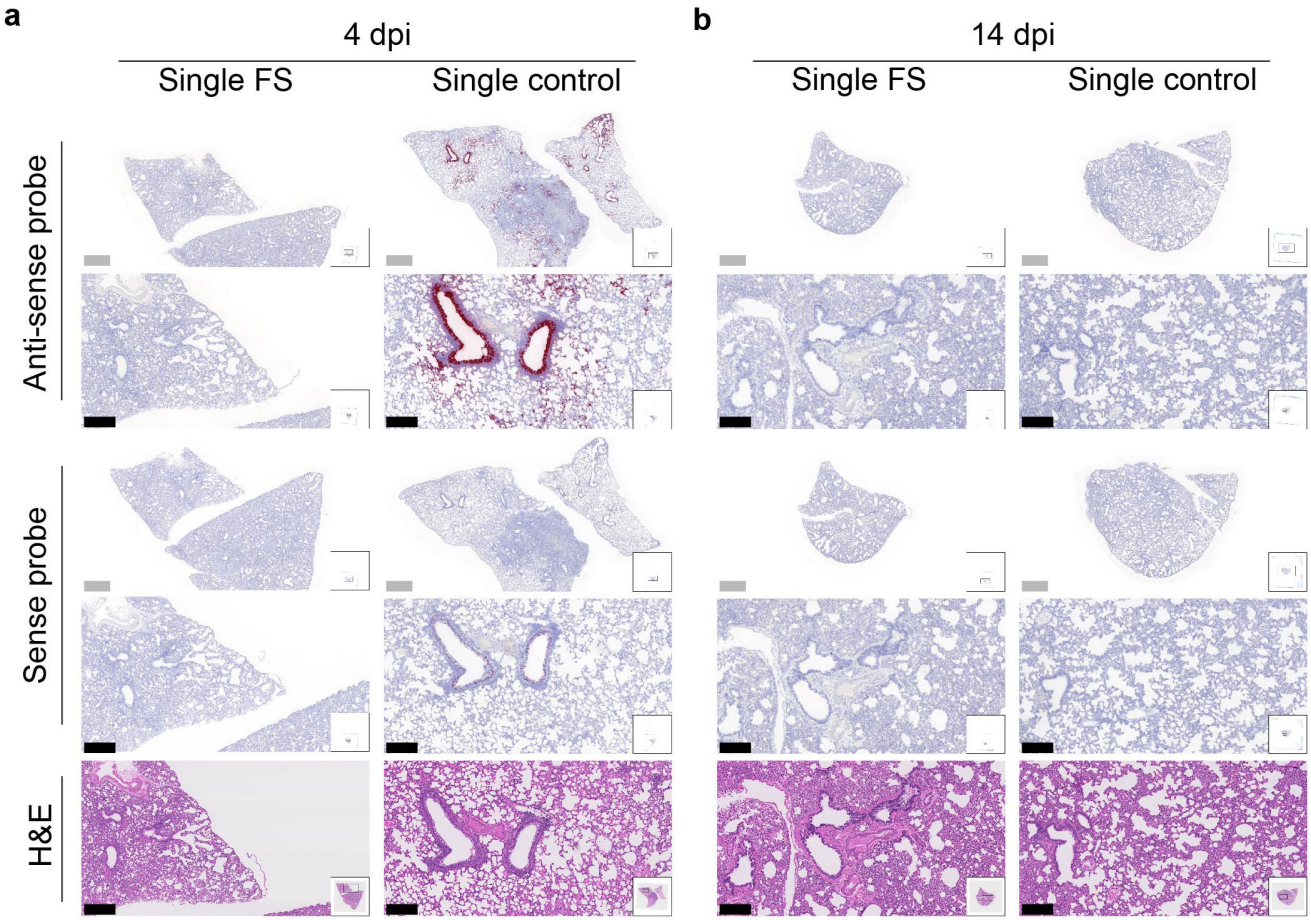
Histopathology of SARS-CoV-2 infection in the lungs of single-dose HD-Ad-FS immunized hamsters. **(A, B)** The presence of SARS-CoV-2 RNA in lung sections was detected with RNAscope *in situ* hybridization at 4 **(A)** and 14 dpi **(B)**. Hematoxylin and eosin staining (H&E, 5th row) was performed with lung sections of the indicated conditions. Continuous lung sections were used for staining. Scale bars = 200 (grey bar) or 1000 (black bar) µm. Images are representatives of n=2 per group. Data represent one independent animal experiment with indicated biological replicates.

The presence and distribution of SARS-CoV-2 in the lungs after challenge was investigated with RNA *in situ* hybridization at 4 and 14 dpi. With the antisense probe, a positive signal was nearly absent in the lungs of single-FS hamsters at 4 dpi, whereas a strong signal was observed in the lungs of single-control hamsters, particularly in bronchial and bronchiolar epithelial cells, alveoli, and interalveolar septa (**Figure 5A**). With the sense probe, the signal was almost undetectable in single-FS hamsters at 4 dpi, but a moderate signal level was observed in single-control hamsters, especially in bronchial epithelial cells (**Figure 5A**). There was no obvious signal observed in the lungs of single-FS and single-control hamsters at 14 dpi (**Figure 5B**). These findings show that single-FS could protect hamsters from SARS-CoV-2 infection and replication in the lung.

### Prime-boost HD-Ad-FS protects the lungs of hamsters from SARS-CoV-2 VOC infection

**Figure 6:**
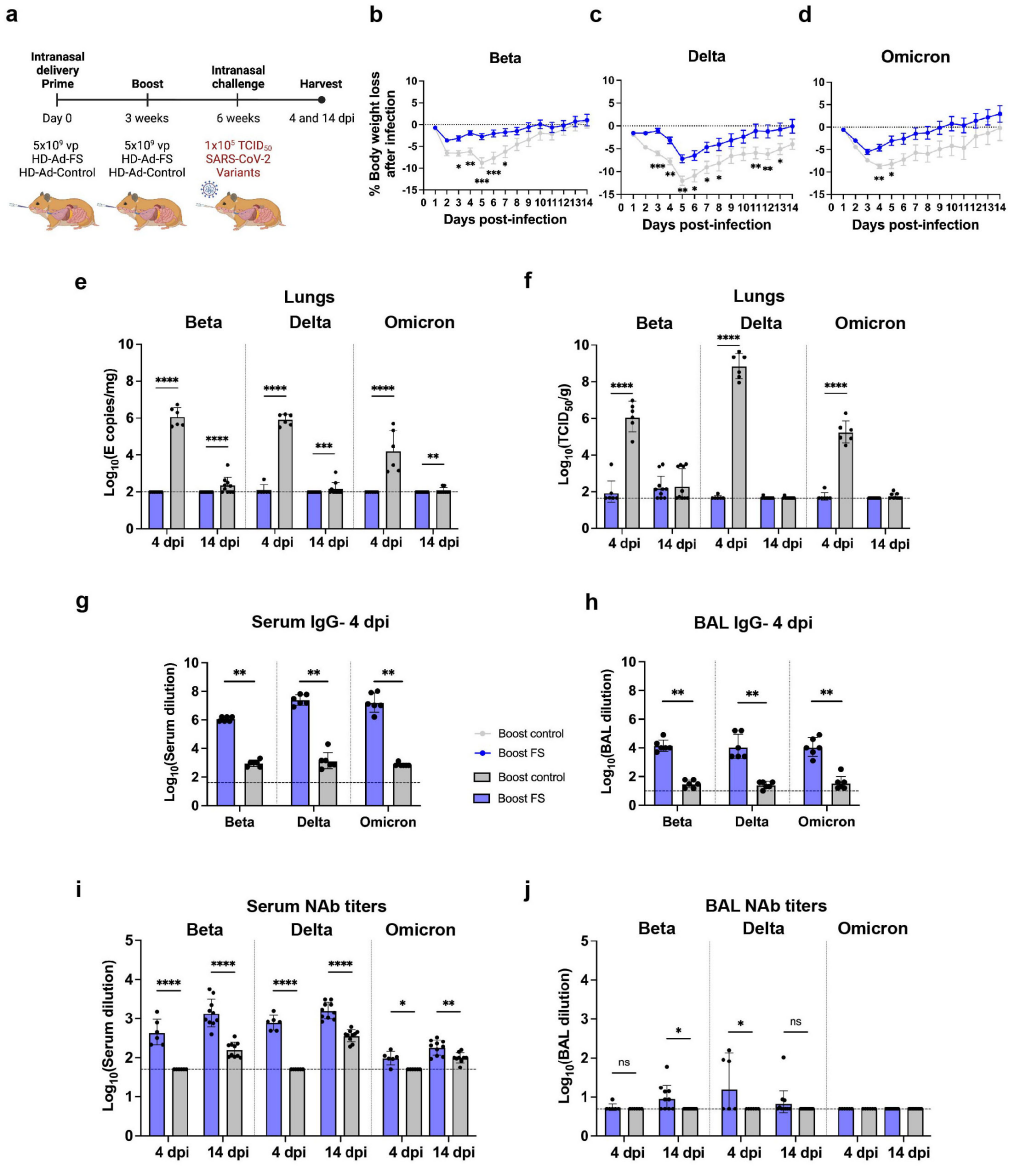
Prime-boost vaccination of HD-Ad-FS protected hamster against SARS-CoV-2 VOCs in the lungs. **(A)** Schematic timeline of the hamster experiment. Hamsters were intranasally immunized with a prime-boost regimen of HD-Ad-FS or HD-Ad-control (5x10^9^ + 5x10^9^ vp, three-week interval). Three weeks after the second dose, the hamsters were intranasally challenged with SARS-CoV-2 variants (Beta, Delta, or Omicron) at 1x10^5^ TCID_50_. **(B-D)** Hamster body weight was monitored at the indicated days after challenging with SARS-CoV-2 variant Beta **(B)**, Delta **(C)**, or Omicron **(D)**. **(E)** RNA levels of SARS-CoV-2 variants in the lungs were determined with RT-qPCR at 4 and 14 dpi. **(F)** Levels of infectious SARS-CoV-2 variants in the lungs were measured with TCID_50_ assay at 4 and 14 dpi. **(G)** The titers of FS-specific IgG in sera were measured with ELISA at 4 dpi. The starting dilution factor was 1:40. **(H)** The titers of FS-specific IgG in BALs were measured with ELISA at 4 dpi. The starting dilution factor was 1:10. **(I)** Serum NAbs against SARS-CoV-2 variants were measured with neutralization assays at 4 and 14 dpi. **(J)** BAL NAbs against the SARS-CoV-2 variants were determined with neutralization assay at 4 and 14 dpi. Dots represent individual hamsters (4 dpi, n=6; 14 dpi, n=9 or 10). For **(E-J)**, the horizontal dotted lines represent the LOD of the assays. For **(B-D)**, statistical analyses were performed by two-way ANOVA; error bars represent mean ± s.e.m. For **(E, F)**, statistical analyses were performed by two-way ANOVA; error bars represent geometric mean with geometric SD. For **(G-J)**, statistical analyses were performed by Mann-Whitney test, two-tailed; error bars represent geometric mean with geometric SD. *p<0.05, **p<0.01, ***p<0.001, ****p<0.0001, and ns, not significant. Data represent one independent animal experiment with indicated biological replicates.

After evaluating the protective efficacy of single-dose HD-Ad-FS, we further investigated the efficacy of prime-boost HD-Ad-FS against the SARS-CoV-2 VOCs Beta, Delta, and Omicron. Hamsters (n=96, equal ratio of sex) were intranasally immunized with a prime-boost dose (5x10^9^ + 5x10^9^ vp) of HD-Ad-FS (boost-FS) or HD-Ad-control (boost-control) at a three-week interval (**Figure 6A**). Three weeks after the boost dose, the hamsters were divided into three groups (n=32/group) and intranasally challenged with Beta, Delta, or Omicron at 1x10^5^ TCID_50_. Due to a housing issue, one hamster (from the Omicron group) died prior to the challenge. At 4 (n=12/group) and 14 (n=19 or 20/group) dpi, the hamsters were euthanized, and serum, BAL, and lung samples were collected and analyzed.

The body weight of hamsters was monitored daily after infection and compared to their pre-infection weight. Both boost-FS and boost-control hamsters experienced weight loss after VOC challenge (**Figures 6B, C, D**). However, the boost-FS hamsters had significantly less weight loss compared to the boost-control hamsters in each group (**Figures 6B–D**). These findings suggest that the HD-Ad-FS could alleviate the morbidity caused by VOC infection in hamsters. Sex-based analysis showed that hamsters of the same sex in the boost-FS group had significantly less weight loss than hamsters in the boost-control group after challenge (**Supplementary Figure 6**).

The levels of viral RNA in the upper airway were measured from oropharyngeal swabs with RT-qPCR. At 4 dpi, the viral RNA levels in the swabs from boost-FS hamsters were significantly lower than those of the boost-control hamsters in each group (**Supplementary Figure 7**). However, viral RNA remained at high levels from 4 to 14 dpi in boost-FS hamsters (**Supplementary Figure 7**).

The levels of viral RNA and infectious virus in the lungs were measured with RT-qPCR and TCID_50_ assays, respectively, at 4 and 14 dpi. At 4 dpi, viral RNA was either undetectable or at very low levels (<10^2^ copies/mg) in boost-FS hamsters, while it was at high levels (>10^4^ copies/mg) in boost-control hamsters in each group (**Figure 6E**). Notably, at 14 dpi, viral RNA was still undetectable or at very low levels (<10^2^ copies/mg) in boost-FS hamsters (**Figure 6E**). Moreover, the infectious virus was undetectable or at very low levels in boost-FS hamsters at 4 and 14 dpi (**Figure 6F**). In contrast, the titers of infectious virus were high in boost-control hamsters at 4 dpi (**Figure 6F**). There were no significant differences in the levels of infectious virus between male and female hamsters in each group (**Supplementary Figure 8A**). These results, combined with the results of viral mRNA levels from oropharyngeal swabs, indicate that the boost-FS could protect hamsters from VOC infection and replication in the lungs, although it may not completely prevent infection in the upper airway.

The levels of FS-specific IgG in sera and BALs were measured with ELISA at 4 dpi. The IgG titers were significantly higher in sera and BALs of all the boost-FS hamsters compared to the boost-control hamsters in each group (**Figures 6G, H**). Specially, in boost-FS hamsters, the reciprocal serum IgG GMTs were 1.1x10^6^ (Beta), 2.4x10^7^ (Delta), and 1.6x10^7^ (Omicron, **Figure 6G**). The reciprocal BAL IgG GMTs were 1.4x10^4^ (Beta), 1.3x10^4^ (Delta), and 1.1x10^4^ (Omicron) in boost-FS hamsters (**Figure 6H**).

The titers of NAbs in sera and BALs were measured with TCID_50_ neutralization assays at 4 and 14 dpi. For the serum NAbs, at 4 dpi, elevated titers were only detected in boost-FS hamsters (**Figure 6J**). Specifically, in boost-FS hamsters, the serum NAb titers were 448.4 (Beta), 785.2 (Delta), and 97.4 (Omicron, **Figure 6I**). At 14 dpi, elevated serum NAb titers were detected in both boost-FS and boost-control hamsters, but the NAb titers were significantly higher in boost-FS hamsters than in boost-control hamsters (**Figure 6I**). For BAL NAbs, at 4 dpi, a significantly increased titer was detected only in the Delta group (3/6) of boost-FS hamsters (**Figure 6J**). The levels of serum (**Supplementary Figure 8B**) and BAL (**Supplementary Figure 8C**) NAbs were significantly higher in female hamsters than male hamsters in the Delta group of boost-FS hamsters at 4 dpi.

**Figure 7:**
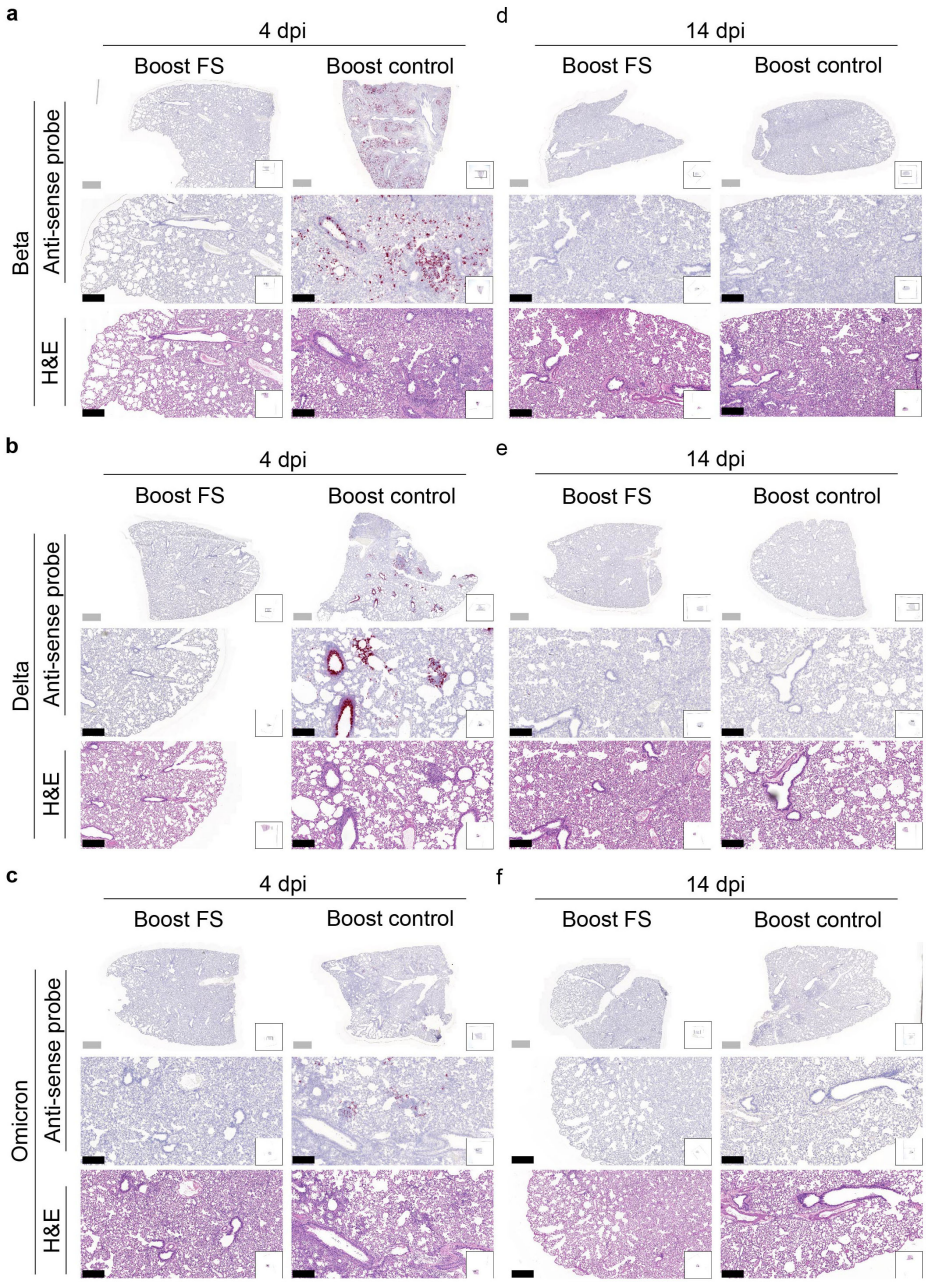
Histopathological analysis of SARS-CoV-2 VOC infection in the lungs of prime-boost HD-Ad-FS vaccinated hamsters. **(A-C)** Detection of SARS-CoV-2 variant RNA (Beta, **A**; Delta, **B**; Omicron, **C**) in lung sections with RNA *in situ* hybridization at 4 dpi. **(D-F)** The presence of SARS-CoV-2 variant RNA (Beta, d; Delta, e; Omicron, f) in lung sections with RNA *in situ* hybridization at 14 dpi. Continuous lung sections were used for H&E staining (3^rd^, 6^th^, and 9^th^ rows). Scale bars = 200 (grey bar) or 1000 (black bar) µm. Images are representatives of n=2 per group. Data represent one independent animal experiment with indicated biological replicates.

RNA *in situ* hybridization was conducted to assess the presence of VOCs in the lungs of hamsters at 4 and 14 dpi. With the antisense probe, the signal was nearly absent in the lungs of boost-FS hamsters in each group at 4 dpi (**Figures 7A-C**). Conversely, strong and abundant signal was detected in the lungs of boost-control hamsters at 4 dpi, particularly in bronchial and bronchiolar epithelial cells, alveoli, and interalveolar septa (**Figures 7A-C**). We did not observe obvious signal in the lungs of boost-FS or boost-control hamsters at 14 dpi (**Figures 7D-F**). These findings suggest that boost-FS could inhibit VOC infection and replication in the lungs of hamsters.

## Discussion

The present study evaluates the efficacy of a HD-Ad-based COVID-19 vaccine candidate (HD-Ad-FS) through intranasal administration in mice and hamsters. The HD-Ad-FS encodes the full-length spike protein of SARS-CoV-2. Following a single-dose of HD-Ad-FS, immunity against the ancestral SARS-CoV-2 strain was induced systemically as well as in the upper and lower airways. We detected potent NAb levels in sera, as well as robust levels of NAb and sIgA on the airway mucosal surface, the first line of defense against viral entry. Notably, minimal or undetectable levels of infectious virus were found in the upper and lower airways after SARS-CoV-2 challenge. In addition, the prime-boost elicited systemic and lower airway protection against SARS-CoV-2 VOCs in hamsters. Compared to our previous HD-Ad-RBD vaccine (23), the HD-Ad-FS vaccine demonstrates superior humoral and cellular immune responses. In particular, HD-Ad-FS generated higher levels of NAb in the airways and elicited an increased number of spike-specific IFN-γ^+^ CD4^+^ and IFN-γ^+^ CD8^+^ T cells in the lungs.

The expression and distribution of vaccine-induced NAbs can be affected by immunization routes (11). Given that SARS-CoV-2 is primarily airborne, airway mucosal NAbs are crucial for blocking viral entry and transmission. However, current COVID-19 IM vaccines mainly induce serum neutralizing activity, with limited mucosal activity (11). In contrast, intranasal vaccination has the potential to induce both serum and mucosal NAbs. The induction of mucosal NAbs is likely achieved by providing antigen to lung-resident T and B cells (15, 33). Indeed, intranasal COVID-19 vaccines have shown promising mucosal immunogenicity in animal models (34–36). For example, a mouse study using an Ad-based vaccine encoding the Omicron BA.1 spike showed that intranasal immunization induced NAbs against BA.1 in both BALs and sera, while IM immunization only induced NAbs in sera (36). Another study using a chimpanzee Ad-based vaccine demonstrated that intranasal immunization elicited serum NAbs and reduced SARS-CoV-2 infection in the upper airway and lungs compared to IM immunization in hamsters (34).

As mentioned above, intranasal administration of HD-Ad-FS induced FS-specific sIgA in the airway. Indeed, airway mucosal sIgA confers protection against respiratory viruses, such as influenza (37) and SARS-CoV-2 (38). In two human studies, the level of sIgA correlates more strongly with protection against SARS-CoV-2 than the level of serum IgG (39, 40). However, we found that, in prime-boost hamsters and hACE2 mice challenged with VOCs, the levels of VOC mRNA in the upper airways did not differ significantly between vaccinated and unvaccinated animals, whereas the levels of infectious VOCs were undetectable in the lungs of vaccinated animals. These findings indicate that HD-Ad-FS can induce immune protection against VOCs in the lungs but not in the upper airway. The mechanism underlying the varied vaccine efficacies in different parts of the airway is not fully understood. Based on our results, since NAbs were only elicited in the sera of prime-boost vaccinated hamsters, it is likely that serum NAbs are more effective at protecting the lung than the upper airway. Alternately, the T cell response mounted in the lung may be more effective against VOCs than the B cell response in the lung. Supporting this suggestion, the VOC epitopes recognized by CD4^+^ and CD8^+^ T cells are better conserved than are the B cell epitopes (41).

We found that HD-Ad-FS vaccinated hamsters had less weight loss and recovered to the pre-infection weight sooner than unvaccinated hamsters. Interestingly, we also observed that male hamsters experienced more weight loss compared to female hamsters, independent of vaccination status, suggesting that sex differences play a role in the severity of the disease. The impact of sex differences on the severity of COVID-19-related disease has been observed in animal studies (42, 43) as well as in clinical studies (44, 45). Males have a higher percentage of severe disease (45) and a higher fatality rate compared to females (44). Although the exact mechanism determining the sex differences is still unknown, studies indicate that sex differences in the immune responses could be important (42, 45). For example, a study showed that activated CD4^+^ and CD8^+^ T cells were more abundant in the sera of female patients, while male patients had lower levels of these T cells (45). In a study using hACE2 mice, male mice had a higher fatality rate than female mice, with inflammatory cytokines and chemokines significantly increased in the lungs of the male mice (42).

The weight loss caused by the Omicron variant in other hamster studies is significantly less than that observed for the sublineage (**Supplementary Table 1**) used in our work (46–48). Given that the challenge dose used in our study was lower than those used in the aforementioned studies, we speculate that the difference in Omicron induced weight loss could be attributed to the varying infectivity of different Omicron sublineages.

Clinical studies have provided evidence supporting the safety of intranasal delivery of Ad-based vaccines (49, 50). For instance, a study demonstrated that intranasal delivery of the prime-boost Ad5-based COVID-19 vaccine was well-tolerated in adults (50). Another vaccine, Ad5-nCoV, using the same Ad5 capsid as used in this study, has been approved for use in the COVID-19 pandemic from the World Health Organization (51, 52). Recently, an Ad-based intranasal vaccine, iNCOVACC (phase III trial), has demonstrated promising mucosal immunogenicity, particularly in inducing an sIgA response, and has been granted emergency use authorization in India (53).

The safety of the HD-Ad vector has been established in clinical trials (54). Our group has demonstrated the safety of delivering an HD-Ad vector into the airways using a porcine model (21). We have also demonstrated that a HD-Ad vaccine encoding the RBD of SARS-CoV-2 was safe in mice (23). Here, we extended this finding and demonstrated the safety of HD-Ad-FS in mice and hamsters. Studies have indicated that the SARS-CoV-2 spike protein could potentially cause tissue damage and adverse effects (55, 56). We recognize the importance of comprehensively evaluating the safety profile of HD-Ad-FS vaccines in future studies.

There are limitations in this study. Firstly, the unexpected mortality during anesthesia limited the sample size for the prime-boost hACE2 mice, thereby reducing the statistical power. Secondly, BAL samples from the single-dose hACE2 mice and hamsters were not collected, which precluded a comprehensive assessment of vaccine-induced mucosal immune responses. Thirdly, we did not measure CD4^+^ and CD8^+^ tissue-resident memory T cells, which are important in providing rapid response and long-term immunity (57). Additionally, while BALB/c, hACE2, and hamsters were used to assess vaccine efficacy, we appreciate that the immune systems of animals do not precisely represent those of humans. Future research should incorporate nonhuman primates that closely mimic humans. Furthermore, we did not determine the profile of the proinflammatory cytokines and chemokines in lung samples. However, we do not expect high levels of vector-induced proinflammatory cytokines and chemokines since the vector dosage used in this study is 3 to10-fold lower than that used for gene delivery (58). Lastly, this study was conducted over a relatively short duration. A long-term study is needed to monitor vaccine-induced immunity over time.

In conclusion, our study demonstrates the safety and efficacy of an intranasal HD-Ad-FS vaccine in eliciting protective systemic and mucosal immunity against SARS-CoV-2 and VOCs. SARS-CoV-2 is continuously evolving to generate new variants and multivalent vaccines are one approach to addressing this issue (59, 60). With the large capacity (36kb) of the HD-Ad vector, multivalent HD-Ad can be engineered to express multiple variant-specific spike antigens simultaneously. Such multivalent HD-Ad vaccines have the potential to induce broadly neutralizing activities and provide protection in regions where multiple variants are circulating. In addition to SARS-CoV-2, other pathogens, including influenza virus, respiratory syncytial virus, human immunodeficiency virus, and rotavirus, rely on penetrating mucosal barriers for infection. Considering the ability of HD-Ad-FS to generate mucosal immunity, HD-Ad is a promising vaccine platform for these transmitted pathogens. Further research is needed to evaluate the efficacy of HD-Ad as vaccines against evolving SARS-CoV-2 variants and other mucosal pathogens.

## Materials and methods

### Tissue culture

Vero E6 and Vero-TMPRSS2 cells were cultured in Dulbecco’s Modified Eagle’s Medium (DMEM, Sigma-Aldrich, D5796) supplemented with 10% heat-inactivated fetal bovine serum (FBS, Gibco, 12483-020) and 1% penicillin/streptomycin (Sigma-Aldrich, P4333). For the Vero-TMPRSS2 cells, blasticidin (A11139-03, Gibco) was added into DMEM at a final concentration of 5 µg/mL. The cells were maintained in T175 tissue culture flasks (Sarstedt, 83.3912.002) at 37°C with 5% CO_2_.

### SARS-CoV-2 strains

The SARS-CoV-2 ancestral (NR-53565), Alpha (B.1.1.7, NR-54011), Beta (B.1.351, NR-54009), Gamma (P.1, NR-54982), Delta (B.1.617.2, NR-55672), and Omicron (BA.1, NR-56461) strains were obtained from BEI resources. The Omicron strain was later confirmed as BA.1.18 by whole-genome sequencing. The ancestral, Alpha, Beta, and Gamma strains were cultured using Vero E6 cells, and the Gamma and Omicron strains were cultured using Vero-TMPRSS2 cells. The full sequences of all strains were confirmed by next-generation sequencing at the Lunenfeld-Tanenbaum Research Institute. All SARS-CoV-2-related experiments were approved by the Institutional Biosafety Committee and performed in Containment Level 3 facilities at EPIC (Emerging & Pandemic Infections Consortium) center of the University of Toronto.

### Animals

BALB/c mice, K18-hACE2 C57BL/6 mice, and hamsters were purchased from the Jackson Laboratory. Mice and hamsters aged between 8-12 weeks were used in the study. Mice were housed in groups. Female hamsters were housed individually, and male hamsters were housed in pairs. All animals were acclimated for at least one week before experiments. The mouse and hamster models were randomly assigned into groups. All animal experiments were approved by the University of Toronto Animal Care Committee or the Hospital for Sick Children Animal Care Committee. Efforts were made to minimize animal suffering. All procedures were performed under anesthesia with isoflurane. The sample size was selected based on our previous study and other relevant studies (23, 32, 61). Ethical guideline on the numbers of animals were followed in designing this study. The sample size represents the minimum number of animals required to address our research question.

### Construction of HD-Ad-FS vaccine and HD-Ad-C4 control

The pC4HSU-NM was used as the backbone for cloning HD-Ad-FS vector. The pPBCMV-sipke-1-1273-S-2P-WPRE contained the FS of SARS-CoV-2. A Bluescript plasmid containing chicken beta actin (CBA) promoter-Ubiquitin C (UbC) intron-bovine growth hormone (bGH) polyadenylation tail was used as a shuttle plasmid. The FS gene was first inserted between the UbC intron and bGH poly(A) tail in the shuttle plasmid by using the Rapid DNA Ligation kit (Thermo Scientific, K1422). Then, the cassette of CBA promoter-UbC intron-FS-bGH poly(A) was cut out from the shuttle plasmid by AscI restriction enzyme and ligated with AscI-digested pC4HSU-NM. The final HD-Ad-FS vector was constructed as inverted terminal repeats (ITR)-CBA promoter-UbC intron-FS-bGH poly(A)-ITR. The pC4HSU-NM was used for constructing HD-Ad-control.

### HD-Ad production

The HD-Ad vectors were produced as previously described by Ng et al. (62). Briefly, the HD-Ad vectors were amplified in producer 116 cells with NG163 helper virus. The NG163 helper virus provided all the essential genes for HD-Ad production, and its package signal was flanked by two loxP sites. The 116 cells expressed Cre recombinase which cleaved off the package signal of NG163 helper virus. As a result, only HD-Ad vector could be packaged. After serial passages, the HD-Ad vectors were harvested from cell lysates and purified by 3 rounds of CsCl density gradient centrifugation. The number of HD-Ad particles were calculated by measuring the absorbance at 260nm.

### Immunization of BALB/c mice

Female BALB/c mice were intranasally immunized with 5x10^9^ vp of either HD-Ad-FS or HD-Ad-control in 20 µl of PBS with 40 µg/mL of diethylaminoethyl (DEAE)-Dextran (Sigma-Aldrich, 30461) and 0.1% L-α–lysophosphatidylcholine (Sigma-Aldrich, L1381). For the single-dose group, mice only received one dose of HD-Ad-FS or HD-Ad-control and were euthanized at day 21 post-vaccination (dpv). For the prime-boost group, mice received two doses of HD-Ad-FS or HD-Ad-control (three-week interval) at the same viral particle dose and were euthanized at 21 dpv after the second dose. Lung, blood, and bronchoalveolar lavage (BAL) samples were collected and stored at -80°C.

### Immunization and viral challenge of K18-hACE2 C57BL/6 mice

K18-hACE2 C57BL/6 mice (24 males and 24 females) were intranasally immunized with 5x10^9^ vp of either HD-Ad-FS or HD-Ad-control in 20 µl of PBS with 40 µg/mL of DEAE-Dextran and 0.1% LPC. For the single-dose group, K18-hACE2 mice (6 males and 6 females) received one dose of HD-Ad-FS or HD-Ad-control. For the prime-boost group, K18-hACE2 mice (18 males and 18 females) received two doses of HD-Ad-FS or HD-Ad-control (three-week interval) at the same viral particle dose. Three weeks after the last vaccination, all K18-hACE2 mice were intranasally challenged with 1x10^5^ TCID_50_ of virus in 50 µl of DMEM. The single-dose group was infected with the ancestral strain, and the prime-boost group was infected with Beta, Delta, or Omicron variants. Body weights were monitored and recorded daily at 0 (before infection) and 4 dpi. The K18-hACE2 mice were euthanized at 4 dpi. Lung, BAL, blood, spleen, and heart samples were collected and stored at -80°C.

### Immunization and viral challenge of hamsters

Hamsters (64 males and 64 females) were intranasally immunized with 5x10^9^ vp of either HD-Ad-FS or HD-Ad-control in 100 µl of PBS with 40 µg/mL of DEAE-Dextran and 0.1% LPC. For the single-dose group, hamsters (16 males and 16 females) received one dose of HD-Ad-FS or HD-Ad-control. For prime-boost group, hamsters (48 males and 48 females) were immunized with two doses of either HD-Ad-FS or HD-Ad-control (three-week interval). Three weeks after the last vaccination, the single-dose hamsters (16 males and 16 females) were intranasally infected with 1x10^5^ TCID_50_ of the ancestral strain, and the prime-boost hamsters (16 males and 16 females for each strain) were intranasally infected with 1x10^5^ TCID_50_ of either Beta, Delta, or Omicron variants in 100 µl of DMEM. Body weights were monitored and recorded daily from 0 (before infection) to 14 dpi. Hamsters were euthanized at 4 (12 hamsters per strain, equal sex ratio) and 14 (20 hamsters per strain, equal sex ratio) dpi. Lung, BAL, blood, spleen, and heart samples were collected and stored at -80°C.

### Protein isolation and Western blot analysis

Proteins were isolated from cell lysates using RIPA buffer (1% Triton X-100, 0.1% SDS, 150mM NaCl, 20mM Tris-HCl, 0.5% Deoxycholate) and 10% proteinase inhibitor (Roche, 11836145001). Protein concentrations were measured by BCA assay (Thermo Fisher Scientific, 23225). The proteins were mixed with 4X Laemmli sample buffer (Bio-Rad, 1610747) and 2.5% beta-mercaptoethanol. 30 ug of protein was incubated at 95°C for 5 minutes before loading onto mini-PROTEAN TGX Stain-Free Gels (Bio-Rad, 4568086). Protein bands were transferred to Amersham Protran Premium 0.45 μm nitrocellulose membranes (GE Healthcare Life Science, 10600003), which were then probed with the SARS Spike protein antibody (Novus Biologicals, NB-56578) at 1:500, and the goat anti-rabbit IgG (H+L)-HRP conjugate antibody (Bio-Rad, 170-6515) at 1:10000. The antibodies were prepared in a blocking solution containing 2.5% albumin (BioShop, ALB001.500) and 2.5% non-fat dry milk (Bio-Rad, 1706404XTU) in TBST (1X TBS + 0.5M Tween-20). A purified recombinant FS protein was used as a positive control.

### Neutralization assay

Vero E6 or Vero-TMPRSS2 cells were seeded into 96-well tissue culture microplates (Corning, 3997) at 20,000 to 30,000 cells/well. Serum and BAL samples were heat-inactivated at 56°C for 30 minutes. The starting dilution ratios for serum and BAL samples were 50-fold and 5-fold, respectively. Subsequently, the samples were serially diluted in DMEM at a 2-fold ratio. Each dilution was performed in 6 technical replicates. The diluted samples were mixed and incubated with 200 TCID_50_ of SARS-CoV-2 or its variants at 37°C for 1 hour. After incubation, the sample-virus mixtures were added into Vero E6 or Vero-TMPRSSII cells. The final DMEM medium contained 2% FBS. Cytopathic effect (CPE) was monitored on day 4 and day 7 using a light microscope (EVOS FLoid Imaging system). The neutralization titer was determined as the highest dilution that protected 50% of cells from infection by SARS-CoV-2 and its variants.

### Measurement of infectious SARS-CoV-2 and variant titers from lung homogenates

The infectious viral titers were determined by TCID_50_ assay. Vero-E6 or Vero-TMPRSS2 cells were seeded into 96-well tissue culture microplates at 20,000 to 30,000 cells/well, 16 hours before inoculation. One lobe of lung tissue was weighted and placed in 1 mL of DMEM. The lobes were homogenized using 5 mm stainless steel beads (Qiagen, 69989) in a homogenizer (Bead Ruptor 4, Omni). Debris was removed by centrifuging the homogenates at 3,000 g for 5 minutes. The supernatants were serially diluted at a 10-fold ratio in DMEM and added to either Vero-E6 or Vero-TMPRSS2 cells in 6 technical replicates. The final DMEM medium contained 1% FBS. The CPE was monitored on day 4 and day 7. The TCID_50_ was calculated by Karber method (63) and normalized to tissue weight.

### Measurement of SARS-CoV-2 and variant viral RNA from lung homogenates and oropharyngeal swabs

For lung homogenates, one lobe of lung tissue was weighted and homogenized with 5 mm stainless steel beads (Qiagen, 69989) in buffer RLT (Qiagen, 79216) using a homogenizer. The RNA was extracted with the RNeasy Mini kit (Qiagen, 74106) following the manufacturer’s instruction, and eluted in 35 µl of water.

For oropharyngeal swabs, samples were collected by oropharyngeal swabbing. In K18-hACE2 mice, swab samples were collected at 0 (post-infection) and 4 dpi. In single-dose hamsters, samples were collected at 1, 4, 6, 8, 11, and 14 dpi. In prime-boosted hamsters, swab samples were collected on 1, 2, 4, 10, and 14 dpi. All samples were stored in 500 µl of PBS at -80°C. Swabs were vortexed and centrifuged before RNA extraction. For each swab sample, 140 µl of eluted PBS was used for RNA extraction by the QIAamp Viral RNA Mini Kit (Qiagen, 52906), as per manufacturer’s instruction. The RNA was eluted in 60 µl of buffer AVE.

The viral RNA (envelop gene) was reverse transcribed and amplified with the Luna Universal One-Step RT-qPCR kit (NEB, E3005). 1 µl of lung RNA and 5 µl of swab RNA were used for the RT-qPCR. Primers (E_Sarbeco_F1 Forward Primer, 10006889; E_Sarbeco_R2 Reverse Primer, 10006891; Integrated DNA Technologies) targeting the envelope (E) gene were used to detect genomic/subgenomic viral RNA. A standard curve was generated by serially diluting the E gene plasmid (2019-nCoV_E_Positive Control, 10006896, Integrated DNA Technologies) at a 10-fold ratio. The viral RNA copies were calculated by converting the Ct values based on the standard curve. The RT-qPCR stages were as follows: 55°C for 10 minutes; 95°C for 1 minute; 95°C for 10 seconds, 58°C for 30 seconds, read plate, 44 cycles; 95°C for 5 seconds; 65°C, 30 seconds; 65°C, 5 seconds, + 0.5°C/cycle, ramp 0.5°C/seconds, read plate, 60 cycles.

### Enzyme-linked immunosorbent assay

96-well microplates (Nunc MaxiSorp flat-bottom, Invitrogen, 44-24-4-21) were coated with purified recombinant FS protein at 1 µg/mL in 50 mM carbonate coating buffer (Thermo Scientific, 28382) at 4°C overnight. Plates were washed with PBST (Phosphate-buffered saline with 0.1% Tween 20) and blocked with blocking buffer (1% BSA in PBST) for 1 hour at room temperature. Serial diluted serum or BAL samples were added to the plates and incubated for 1 hour at 37°C. After washing with PBST, the plates were incubated with goat anti-mouse IgG conjugated with horseradish peroxidase (1:5000, Invitrogen, 31430), goat anti-mouse IgA conjugated with horseradish peroxidase (1:2000, Invitrogen, 62-6720), or goat anti-hamster IgG conjugated with horseradish peroxidase (1:4000, Thermo Scientific, PA1-29626) in blocking solution for 1 hour at room temperature. Signal was developed with 3,3’,5,5’,-tetramethylbenzidine (TMB, Thermo Scientific, 34028) for 1 hour at room temperature. The reaction was stopped by adding 1 M H_2_SO_4_. Plates were read at OD_450_ (Cytation 5, BioTek).

### Flow cytometry analysis

Left lung lobes and spleens from single-FS and single-control immunized BALB/c mice were used for flow cytometry analysis. Lung lobes were digested with a digestion buffer containing 2 ug/mL of Liberase (Roche Diagnostics, 1988433) and 25 unites/mL of type IV DNase I (Sigma-Aldrich, D5025) at 37°C for 45 minutes. Following digestion, lung tissues were dispersed with 18-gauged needles and filtered through 100 nm cell strainers (Falcon, 352360). Spleenocytes were isolated by straining spleens through 70 nm cell strainers (Falcon, 352350). Red blood cells (RBC) were lysed by resuspending filtered cells in 1X RBC Lysis Buffer (eBioscience, 00-4333-57). Subsequently, each cell sample was split into two replicates. One replicate was incubated with purified FS protein at 10 µg/mL at 37°C for 12 hours, while the other replicate was not incubated with FS protein. After incubation, GolgiPlug (Fixation/Permeabilization Solution Kit with BD GolgiPlug, 555028) was added for 6 hours. Cells were stained with the Live/Dead Fixable Violate Dead Cell kit (Invitrogen, L34955) and blocked with CD16 and CD32 antibodies (Mouse BD Fc Block, 553142). Next, cells were stained with surface markers: BV711 rat anti-mouse CD4 (BD Biosciences, 563726), APC-Cy7 rat anti-mouse CD8a (BD Biosciences, 557654), PE-Cy7 rat anti-mouse CD19 (BD Biosciences, 552854), and BV510 rat anti-mouse CD44 (BD Biosciences, 563114). The cells were then fixed and permeabilized with Fixation/Permeabilization Solution Kit (BD Biosciences, 555028). Finally, cells were stained with intracellular markers: FITC rat anti-mouse IFN-γ (BD Biosciences, 554411), and PE rat anti-mouse TNF-α (BD Biosciences, 554419). Flow cytometry was performed on a Becton Dickinson LSR II CF I and analyzed with Flowjo v10 software.

### Histopathology and RNA *in situ* hybridization

Lung tissues were fixed directly in 10% neutral phosphate buffered formalin (Sigma-Aldrich, HT501128) without inflation for a minimum of 2 weeks prior to paraffin embedding. The paraffin blocks were continuously sectioned into 4 μm-thick slices and mounted onto slides (Fisherbrand, 12-550-15). The hematoxylin and eosin (H&E) staining was performed at the Centre for Phenogenomics in Toronto. The RNA *in situ* hybridization was performed using the RNAscope 2.5 High Definition-RED Assay (Advanced Cell Diagnostic, 322350), according to manufacturer’s instruction. The Probe-V-nCoV2019-S (Advanced Cell Diagnostic, 848561) was an anti-sense probe for spike RNA. The Probe-V-nCoV2019-S-sense was a sense probe for spike RNA.

Briefly, formalin-fixed, paraffin-embedded tissues were baked at 60°C for 1 hour and then deparaffinized using xylene and ethanol. Subsequently, samples were treated with hydrogen peroxide for 10 minutes at room temperature, followed by target retrieval using RNAscope Target Retrieval Solution. Samples were incubated with Protease Plus solution at 40°C for 30 minutes. After hybridizing with the probes, signals were detected using a mixture of RED-B and RED-A. A counterstain was performed using 50% hematoxylin and 0.02% ammonia water. The slides were mounted with VectaMount Permanent Mounting Medium (Vector Laboratories, H-5000-60) and cover clips (VWR, 48366-067), and scanned with a 3DHistech Slide Scanner at the Imaging Facility at the Hospital for Sick Children.

### Data analysis

The results and graphs were generated with GraphPad Prism version 8 (GraphPad Software). Statistical analyses were performed using analysis of variance (ANOVA) with multiple comparison test or unpaired t-test (two-tailed) with Mann-Whitney test. ChatGPT (OpenAI, GPT-3, 2023) was employed for grammar checking during the preparation of this manuscript. All AI-assisted revisions were reviewed and approved by the authors to ensure the accuracy and quality of the final manuscript.

## Data Availability

The original contributions presented in the study are included in the article/**Supplementary Materials**. Further inquiries can be directed to the corresponding authors.
