# Supplementary material for: Intranasal HD-Ad-FS vaccine induces systemic and airway mucosal immunities against SARS-CoV-2 and systemic immunity against SARS-CoV-2 variants in mice and hamsters
Source: Front Immunol. 2024 Aug 30;15:1430928. doi: 10.3389/fimmu.2024.1430928 (PMC11392758; doi:10.3389/fimmu.2024.1430928)
Supplement: Supplementary file 1 [file DataSheet1.docx]

**Supplementary Materials for**

Intranasal HD-Ad-FS Vaccine Induces Systemic and Airway Mucosal Immunities Against SARS-CoV-2 and Systemic Immunity against SARS-CoV-2 Variants in Mice and Hamsters

Peter Zhou^1†^, Jacqueline Watt^1†^, Juntao Mai^1^, Huibi Cao^2^, Zhijie Li^1^, Ziyan Chen^2, 5^, Rongqi Duan^2^, Ying Quan^1^, Anne-Claude Gingras^1, 3^, James M. Rini^1, 4*^, Jim Hu^2, 5*^ and Jun Liu^1*^

† Authors contributed equally to this work

*Correspondence: James M. Rini, Tel: 416-978-0557, [james.rini@utoronto.ca](mailto:james.rini@utoronto.ca); Jim Hu, Tel: 416-813-6412, [jim.hu@sickkids.ca](mailto:jim.hu@sickkids.ca); Jun Liu, Tel: 416-946-5067, [jun.liu@utoronto.ca](mailto:jun.liu@utoronto.ca)

**This PDF file includes:**

Supplementary Figure 1-8 with corresponding legends

**
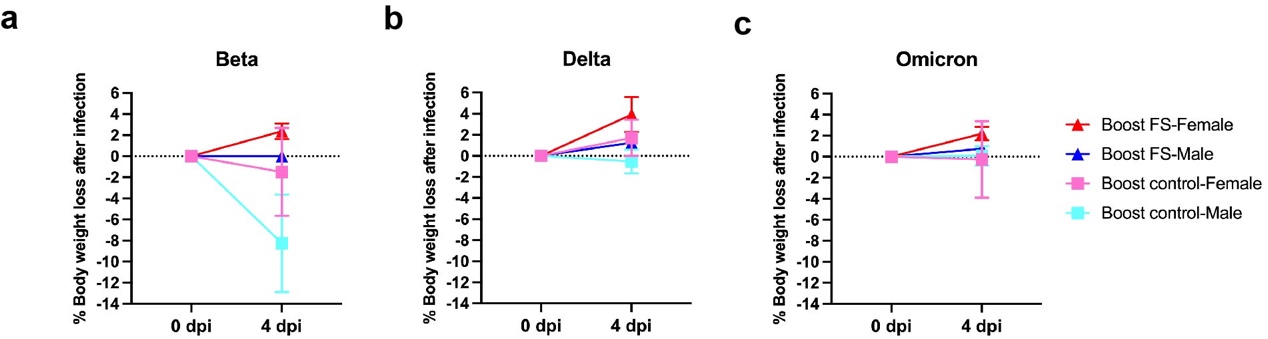
**

**Supplementary Figure 1.** Sex-based analyses of weight change in prime-boost HD-Ad-FS vaccinated hACE2 mice. The vaccinated hACE2 mice were challenged with either Beta **(a)**, Delta **(b)**, or Omicron **(c)** strain. Weight change of the challenged hACE2 mice was monitored at 0 (pre-challenge) and 4 dpi. Dots represented individual hamsters.


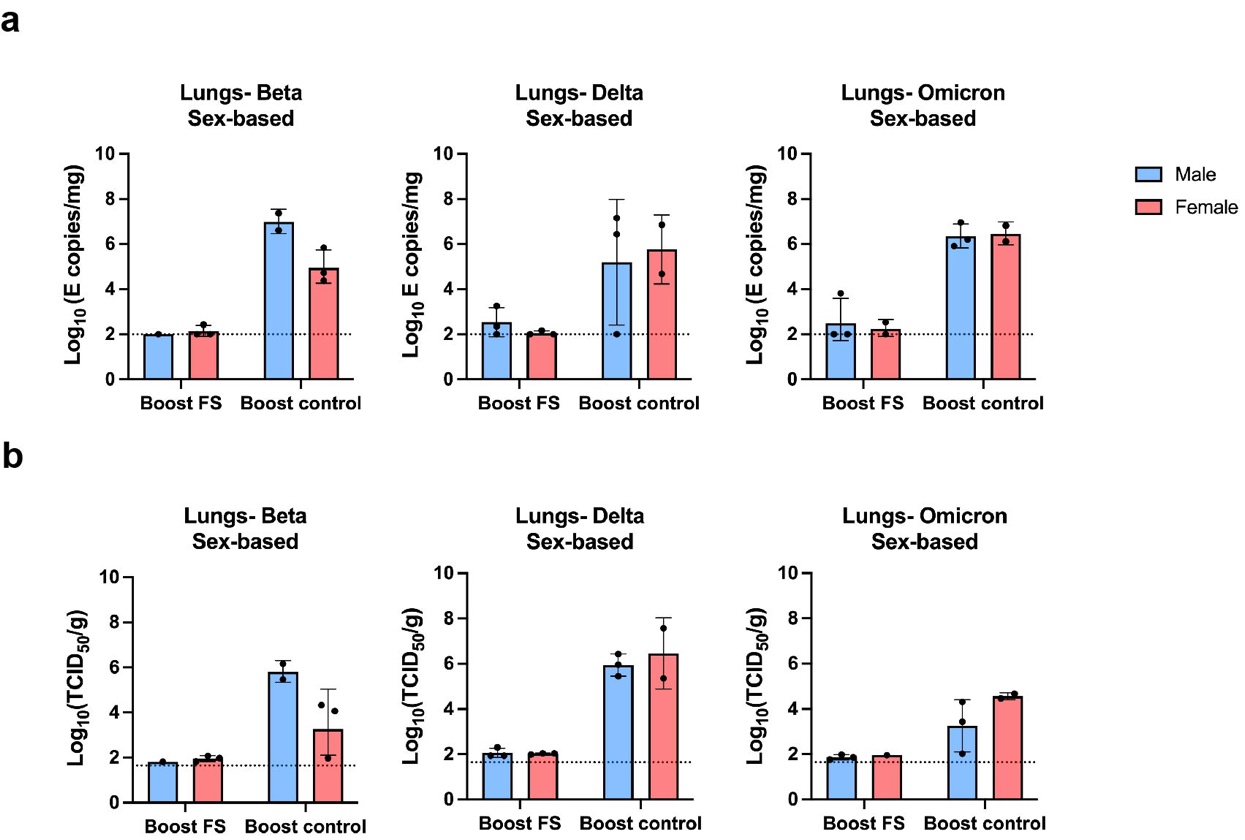


**Supplementary Figure 2. (a)** Sex-based analyses of SARS-CoV-2 variant RNA levels in the lungs of prime-boost immunized hACE2 mice at 4 dpi. The viral RNA was determined with RT-qPCR. **(b)** Sex-based analyses of infectious SARS-CoV-2 variant titers in lungs of prime-boost immunized hACE2 mice at 4 dpi. The titers of infectious virus were determined with TCID_50_ assay. Dots represented individual hamsters. The dotted lines represent the LOD of the assays.


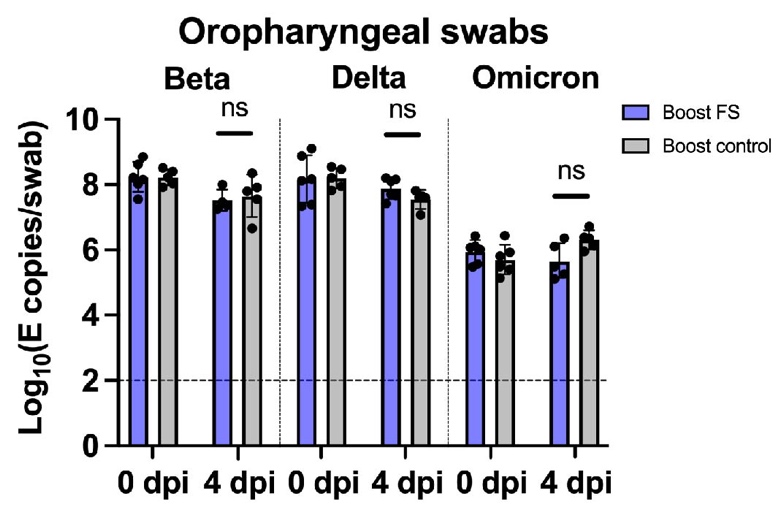


**Supplementary Figure 3.** RNA levels of SARS-CoV-2 variants in oropharyngeal swabs from prime-boost immunized hACE2 mice. Swabs were collected at 0 (post-challenge) and 4 dpi. The RNA levels were determined with RT-qPCR. Dots represent individual hACE2 mice (n=4, 5, or 6). Dots represented individual hamsters. The horizontal dotted lines represent the LOD of the assays. Statistical analysis was performed by two-way ANOVA. Bars and errors represent the geometric mean with geometric SD. ns, not significant.


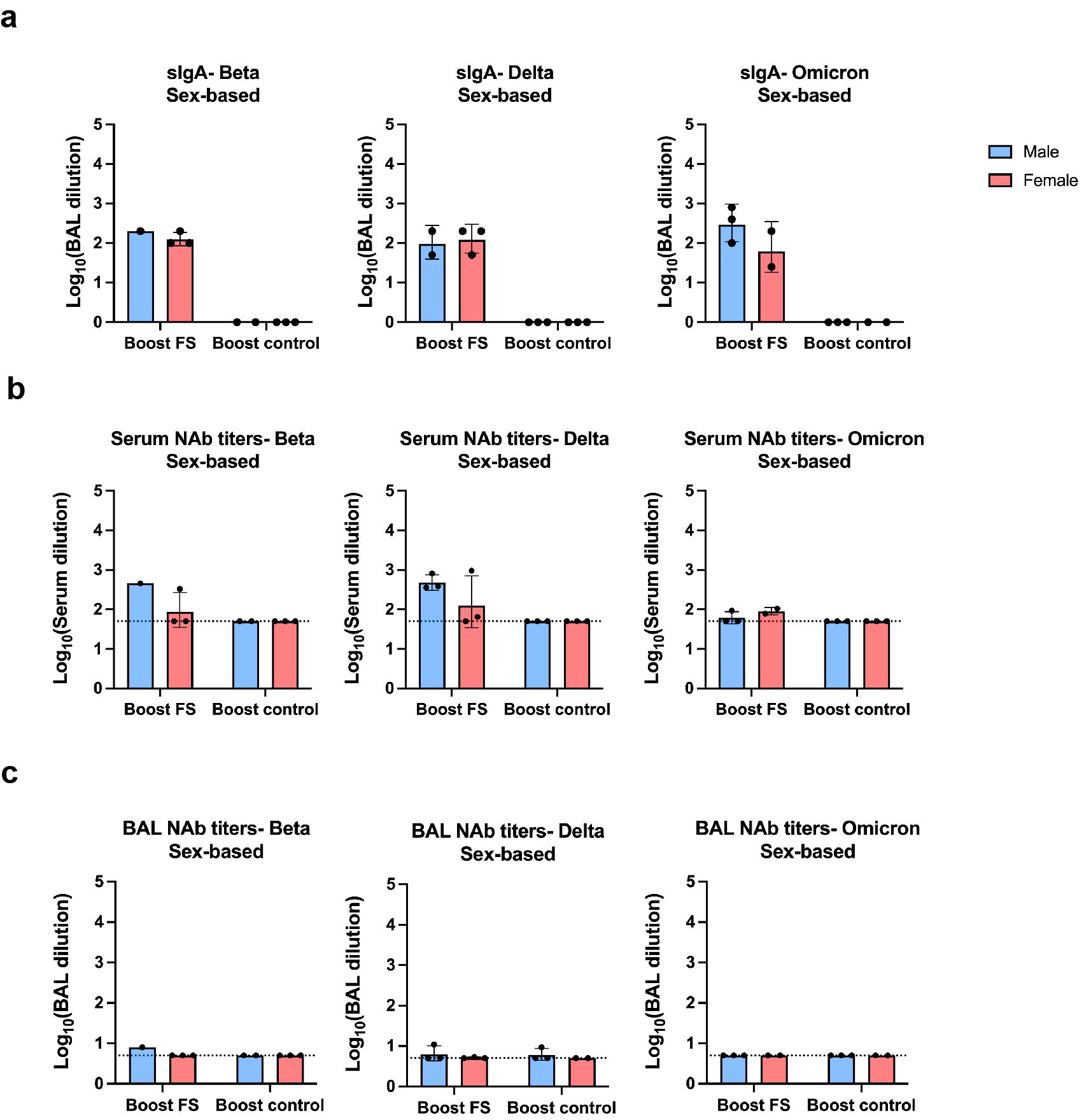


**Supplementary Figure 4. (a)** Sex-based analysis of the levels of FS-specific sIgA in BALs of prime-boost immunized hACE2 mice at 4 dpi. The starting dilution factor was 1:1. **(b)** Sex-based analysis of the levels of serum NAbs in prime-boost immunized hACE2 mice at 4 dpi. **(c)** Sex-based analysis of the levels of BAL NAbs in prime-boost immunized hACE2 mice at 4 dpi. Dots represented individual hamsters. For **(b and c)**, the dotted lines represent the LOD of the assays.


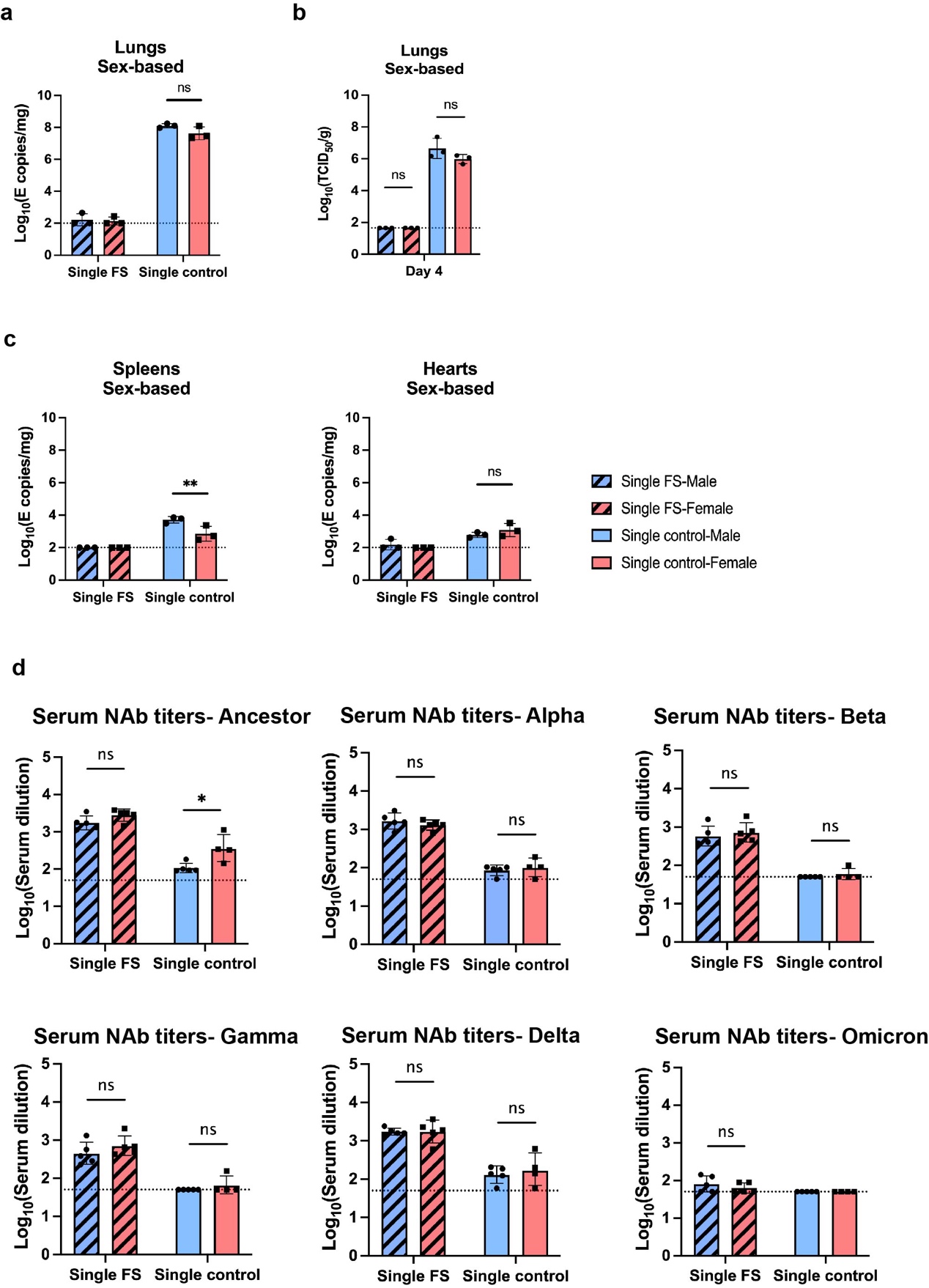


**Supplementary Figure 5. (a)** Sex-based analysis of SARS-CoV-2 RNA levels in the lungs of single-dose immunized hamsters. The viral RNA was determined with RT-qPCR at 4 dpi. **(b)** Sex-based analysis of infectious SARS-CoV-2 titers in lungs of single-dose immunized hamsters. The titers of infectious virus were determined with TCID_50_ assays at 4 dpi. **(c)** Sex-based analysis of SARS-CoV-2 RNA levels in spleens and hearts of single-dose immunized hamsters. The viral RNA was determined with RT-qPCR at 4 dpi. **(d)** Sex-based analysis of serum NAb titers against SARS-CoV-2 and variants. Sera were collected from single-dose immunized hamsters at 14 dpi. Dots represented individual hamsters. The dotted lines represent the limit of detection (LOD) of the assays. Statistical analysis was performed by one-way ANOVA. Error bars represent geometric mean with geometric SD. *p<0.05, **p<0.01, and ns, not significant.


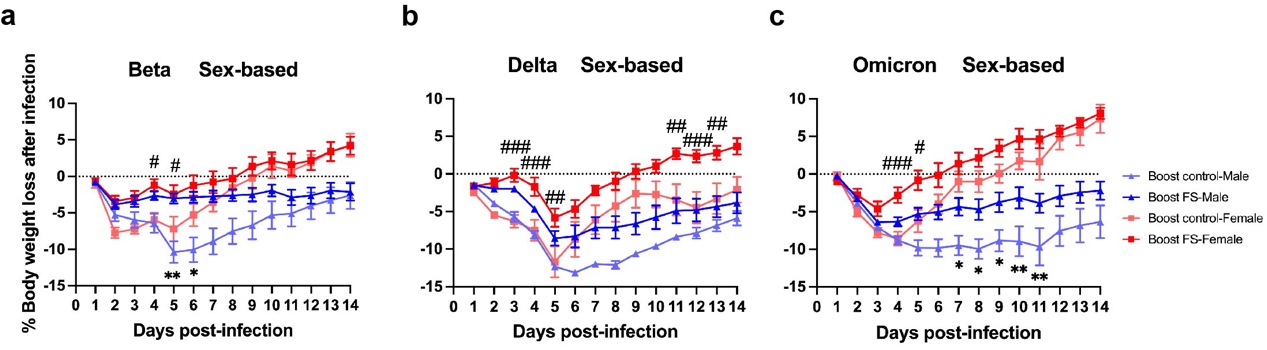


**Supplementary Figure 6.** Sex-based analysis of weight change in prime-boost immunized hamsters. The weight was monitored at the indicated days after challenging with SARS-CoV-2 variant. The asterisk signs represent statistical analysis in boost-FS hamsters, and the pound signs represent statistical analysis in boost-control hamsters. Dots represented individual hamsters. */#p<0.05, **/##p<0.01, ###p<0.001.


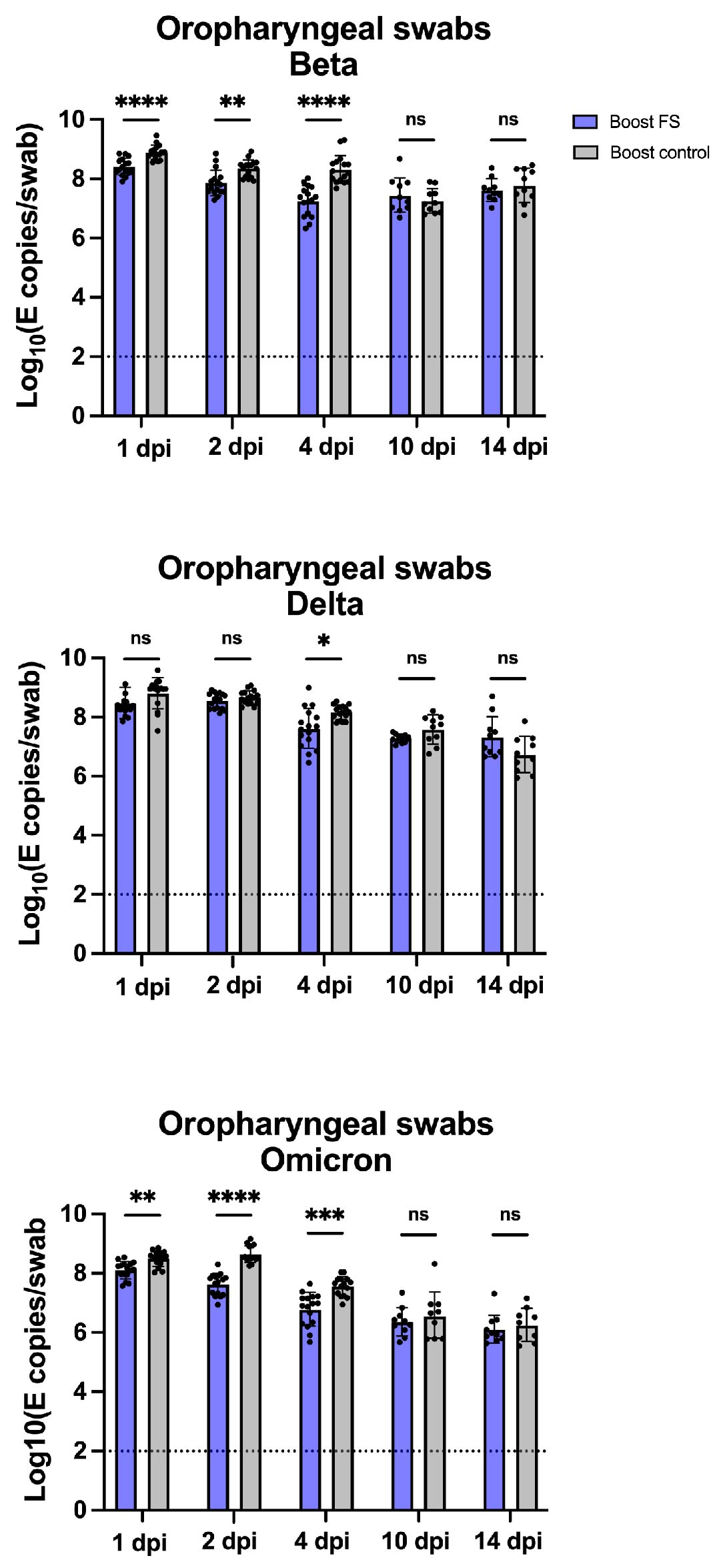


**Supplementary Figure 7.** RNA levels of SARS-CoV-2 variants in oropharyngeal swabs from prime-boost immunized hamsters. Swabs were collected at the indicated time points. The RNA levels were determined with RT-qPCR. Dots represented individual hamsters. The dotted lines represent the LOD of the assays. Statistical analysis was performed by two-way ANOVA. Bars and errors represent the geometric mean with geometric SD. *p<0.05, **p<0.01, ***p<0.001, ****p<0.0001, ns, not significant.


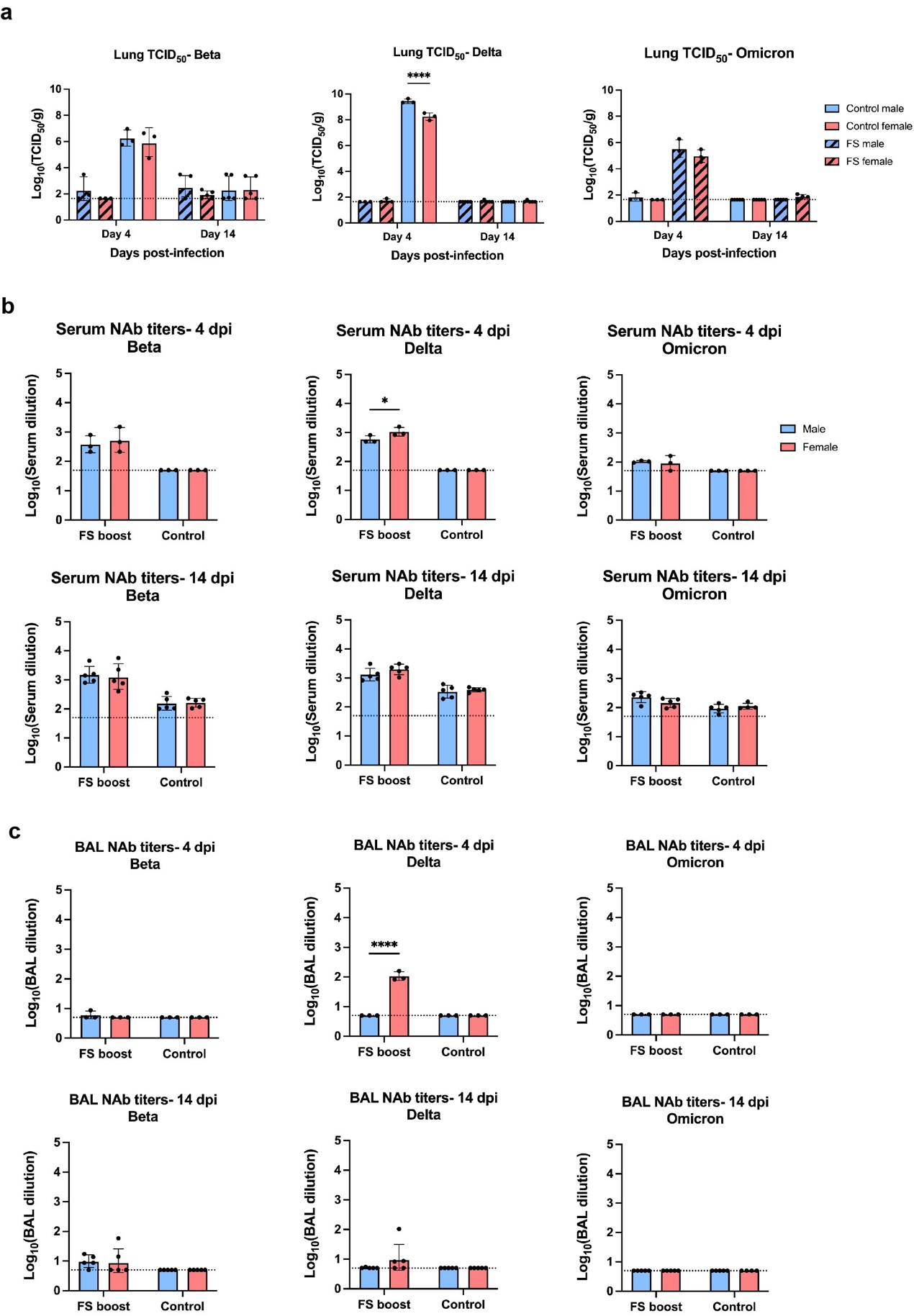


**Supplementary Figure 8. (a)** Sex-based analysis of infectious titers of the SARS-CoV-2 variants in the lungs of prime-boost immunized hamsters. The titers were determined with TCID_50_ assays at 4 and 14 dpi. Statistical analysis was performed by two-way ANOVA. Error bars represent geometric mean with geometric SD. ****, p<0.0001. **(b)** Sex-based analysis of serum NAbs against SARS-CoV-2 variants. Sera of prime-boost immunized hamsters were collected at 4 and 14 dpi. Statistical analysis was performed by one-way ANOVA. Error bars represent geometric mean with geometric SD. *, p<0.05, ns, not significant. **(c)** Sex-based analysis of NAbs in BALs against SARS-CoV-2 variants. BALs of prime-boost immunized hamsters were collected at 4 and 14 dpi. Statistical analysis was performed by one-way ANOVA. Error bars represent geometric mean with geometric SD. ****p<0.0001. Dots represented individual hamsters. The dotted lines represent the LOD of the assays.
